# Postharvest seed coat darkening in pinto bean (*Phaseolus vulgaris*) is regulated by *P^sd^*, an allele of the basic helix‐loop‐helix transcription factor *P*

**DOI:** 10.1002/ppp3.10132

**Published:** 2020-08-19

**Authors:** Nishat S. Islam, Kirstin E. Bett, K. Peter Pauls, Frédéric Marsolais, Sangeeta Dhaubhadel

**Affiliations:** ^1^ London Research and Development Centre Agriculture and Agri‐Food Canada London ON Canada; ^2^ Department of Biology University of Western Ontario London ON Canada; ^3^ Department of Plant Sciences University of Saskatchewan Saskatoon SK Canada; ^4^ Department of Plant Agriculture University of Guelph Guelph ON Canada

**Keywords:** Basic helix‐loop‐helix transcription factor, gene‐specific marker, genetic complementation, pinto bean, postharvest seed coat darkening, proanthocyanidins, transparent testa

## Abstract

Pinto bean (*Phaseolus vulgaris*) is one of the leading market classes of dry beans that is most affected by postharvest seed coat darkening. The process of seed darkening poses a challenge for bean producers and vendors as they encounter significant losses in crop value due to decreased consumer preference for darker beans. Here, we identified a novel allele of the *P* gene, *P^sd^*, responsible for the slow darkening seed coat in pintos, and identified trait‐specific sequence polymorphisms which are utilized for the development of new gene‐specific molecular markers for breeding. These tools can be deployed to help tackle this economically important issue for bean producers.

**Summary:**

- Postharvest seed coat darkening in pinto bean is an undesirable trait that reduces the market value of the stored crop. Regular darkening (RD) pintos darken faster after harvest and accumulate higher level of proanthocyanidins (PAs) compared to slow darkening (SD) cultivars. Although the markers cosegregating with the SD trait have been known for some time, the *SLOW DARKENING* (*Sd*) gene identity had not been proven.
- Here, we identified *P^sd^* as a candidate for controlling the trait. Genetic complementation, transcript abundance, metabolite analysis, and inheritance study confirmed that *P^sd^* is the *Sd* gene. *P^sd^* is another allele of the *P* (*Pigment*) gene, whose loss‐of‐function alleles result in a white seed coat.
- *P^sd^* encodes a bHLH transcription factor with two transcript variants but only one is involved in PA biosynthesis. An additional glutamate residue in the activation domain, and/or an arginine to histidine substitution in the bHLH domain of the *P^sd^‐1* transcript in the SD cultivar is likely responsible for the reduced activity of this allele compared to the allele in a RD cultivar, leading to reduced PA accumulation.
- Overall, we demonstrate that a novel allele of *P*, *P^sd^*, is responsible for the SD phenotype, and describe the development of new, gene‐specific, markers that could be utilized in breeding to resolve an economically important issue for bean producers.

## Graphical abstract

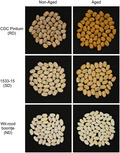

## 1 INTRODUCTION

**FIGURE 1:**
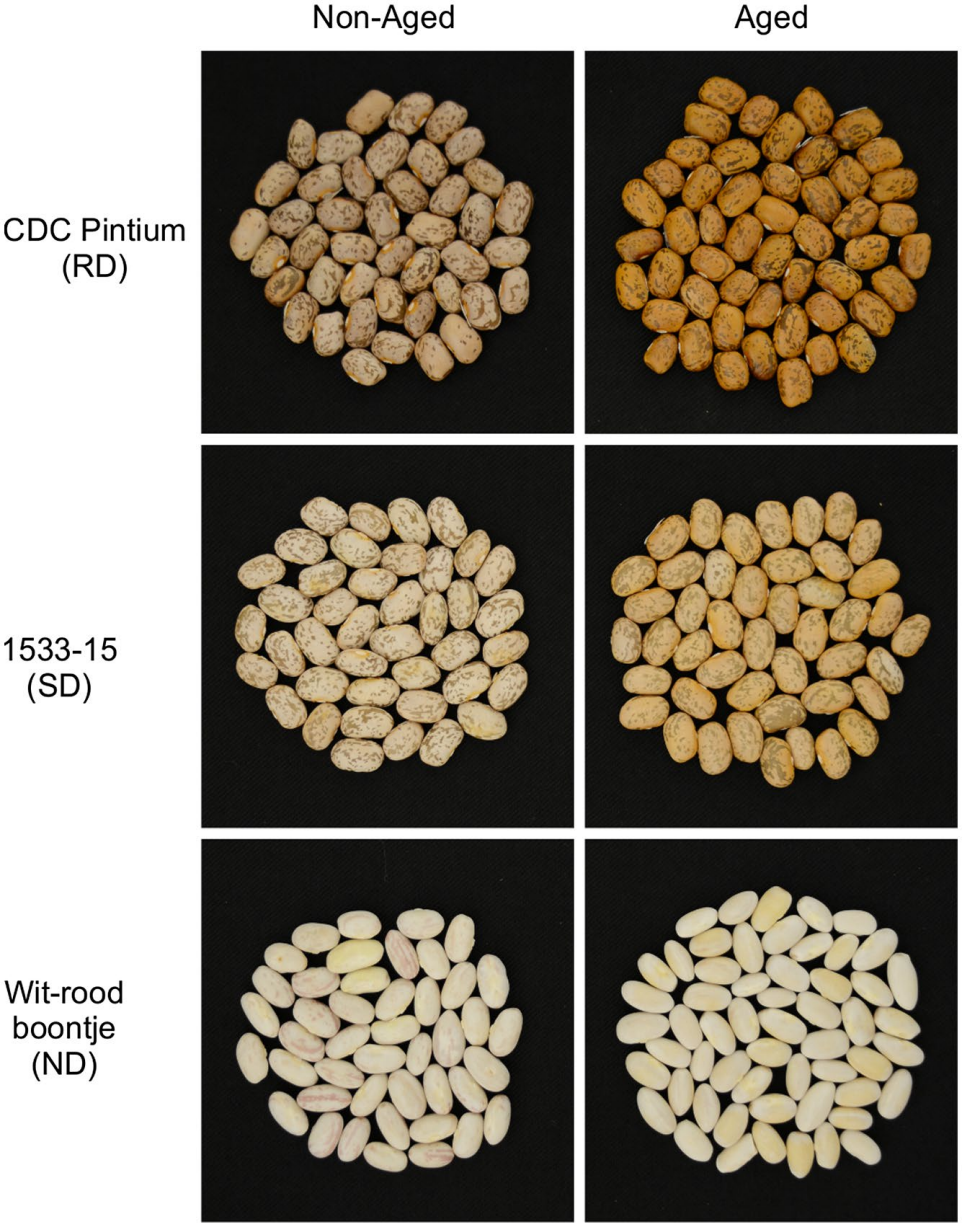
Postharvest seed coat darkening effects in pinto bean. Pictures were taken for pinto bean lines CDC Pintium (RD), 1533‐15 (SD), and cranberry‐like Wit‐rood boontje (ND) on the same day of harvest (non‐aged) and after 6 months of storage at room temperature (aged)

Postharvest darkening of the seed coat is a worldwide problem for the storage and marketing of dry beans (*Phaseolus vulgaris* L.). Many colored beans such as pinto, cranberry, carioca, and light red kidney beans darken faster upon exposure to elevated temperature, humidity, and light (Junk‐Knievel, Vandenberg, & Bett, 2007; Park & Maga, 1999). Furthermore, crop genotype plays an important role in the postharvest seed coat darkening (Elsadr, Wright, Pauls, & Bett, 2011; Junk‐Knievel, Vandenberg, & Bett, 2008). Bean producers and vendors encounter significant losses in crop value due to the decreased consumer preference for the darker beans. Pinto bean is one of the leading market classes of dry beans that suffer most by darkening of seed coat after harvest. Based on the postharvest seed coat color, pinto cultivars are categorized into three groups: (a) fast or regular darkening (RD), (b) slow darkening (SD) where seed coat darkens to a lesser extent than RD, and (c) non‐darkening (ND; Elsadr et al., 2011). Currently, SD cultivars, which can attract a higher premium, are available for producers. As shown in Figure 1, seed coat color in ND cranberry‐like bean Wit‐rood boontje never darkens, whereas it changes from creamy white to brown upon aging in CDC Pintium (RD) and creamy white to stable light brown in 1533‐15 (SD) under the same conditions. CDC Pintium (RD) and 1533‐15 (SD; registered in Canada as CDC WM‐1) are two pinto bean cultivars developed by the Crop Development Centre at the University of Saskatchewan, Canada, that have been used extensively to investigate the postharvest seed coat darkening phenomenon. Segregation analysis using these RD and SD lines revealed that the SD trait is controlled by a single recessive gene, *sd* (Elsadr et al., 2011; Junk‐Knievel et al., 2008). Subsequently, two simple sequence repeat (SSR) markers, Pvsd‐1158 and Pvsd‐1157, located on chromosome 7 that are tightly linked with the SD trait and their linkage distances from *Sd* gene were identified (Felicetti et al., 2012). More recently, a SNP marker within the *Pigment* (*P*) gene that cosegregates with the SD trait, was published (Alvares et al., 2019).

**FIGURE 2:**
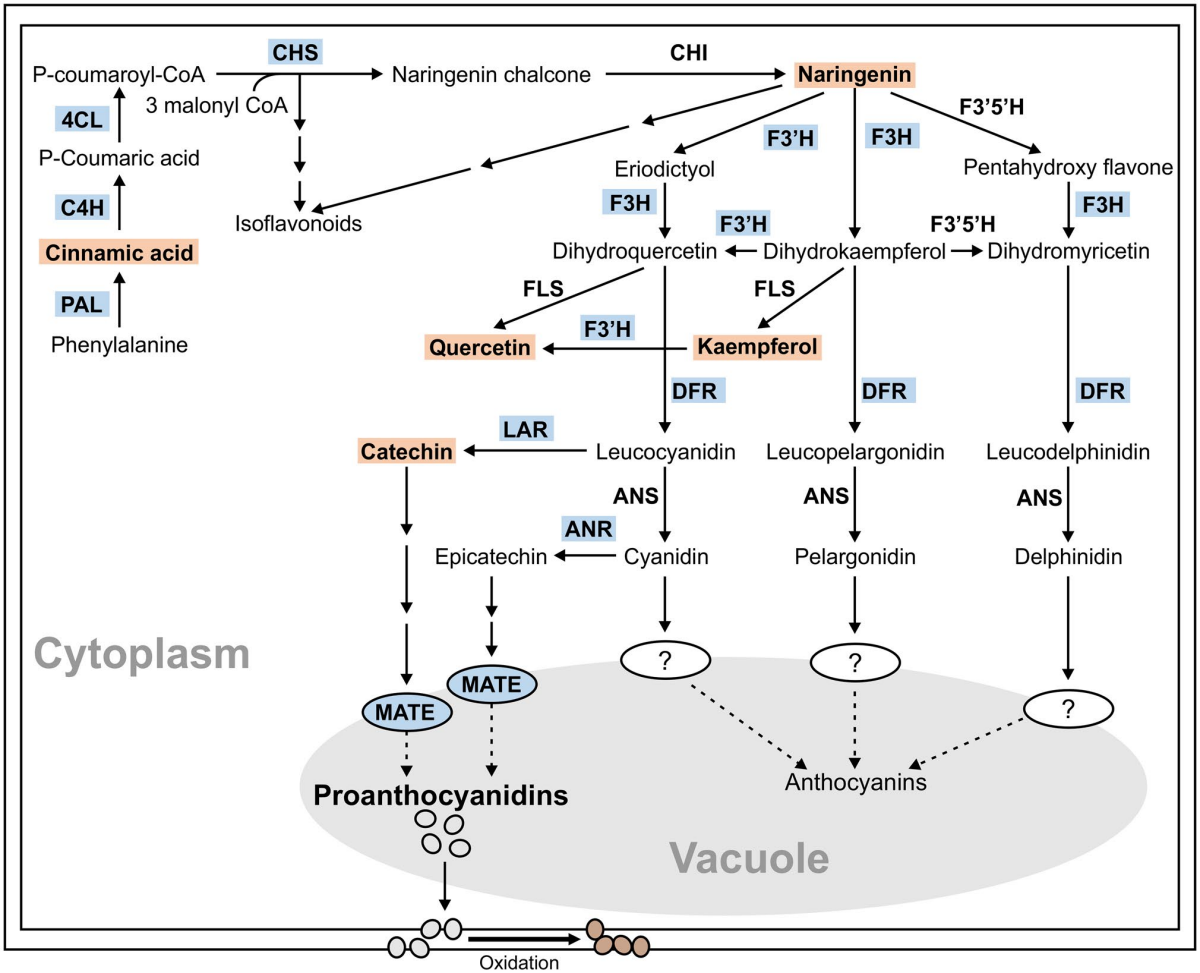
Scheme of proanthocyanidin biosynthesis in pinto bean. The dashed arrows represent speculative steps and multiple arrows indicate multiple steps. The corresponding transcripts (blue highlights) and metabolites (orange highlights) accumulate at higher level in developing seed coats of CDC Pintium (RD) compared to 1533‐15 (SD; Duwadi et al.. 2018). 4Cl, 4‐coumarate‐CoA ligase; ANR, anthocyanidin reductase; ANS, anthocyanidin synthase; ANS, anthocyanidin synthase; C4H, cinnamate‐4‐hydroxylase; CHI, chalcone isomerase; CHR, chalcone reductase; CHS, chalcone synthase; DFR, dihydroflavonol reductase; F3’5’H, flavonoid 3′,5′‐hydroxylase; F3’H, flavonoid 3′‐hydroxylase; F3H, flavanone 3‐hydroxylase; FLS, flavonol synthase; LAR, leucoanthocyanidin reductase; MATE, multidrug and toxic compound extrusion protein; PAL, phenylalanine ammonia lyase

CDC Pintium accumulates larger amounts of proanthocyanidins (PAs) and PA monomers compared to 1533‐15 in both aged and non‐aged seeds (Beninger et al., 2005), and developing seed coat tissues (Duwadi et al., 2018; Elsadr, Marles, Caldas, Blair, & Bett, 2015). PAs are specialized metabolites, abundantly found in seed coat tissue, and are one of the end products of complex flavonoid biosynthetic pathway (Dixon, Xie, & Sharma, 2005). They are oligomers or polymers of flavan‐3‐ol units: (−)‐epicatechin and (+)‐catechin. As shown in Figure 2, PA biosynthesis branches out from leucocyanidin and cyanidin to produce PA monomers catechin and epicatechin, respectively. Glycosylated catechin and epicatechin units are transported from cytosol to the vacuole by a vacuolar multidrug and toxic compound extrusion (MATE) transporter (Zhao & Dixon, 2009), where they polymerize via either an enzymatic or non‐enzymatic process to form colorless PA oligomers (Liu, Wang, Shulaev, & Dixon, 2016). It is speculated that vacuolar PAs are transported via membrane vesicles or some other mechanisms to the apoplastic space, the site of PA oxidation (Zhao, Pang, & Dixon, 2010), where they get oxidized by a laccase‐like enzyme giving rise to a brown color (Pourcel, Routaboul, Cheynier, Lepiniec, & Debeaujon, 2007). Characterization of over 20 *Arabidopsis thaliana transparent testa* (*tt*) mutants, with defects in PA accumulation, has led to an understanding of the structural and regulatory mechanisms of PA synthesis, their modification and transport, and the processes that impact overall PA accumulation (Appelhagen et al., 2014; Shirley et al., 1995; Zhao et al., 2010). Biosynthesis of PA monomers is controlled by the late phenylpropanoid pathway biosynthetic genes: *DIHYDROFLAVONOL 4‐REDUCTASE (DFR), LEUCOANTHOCYANIDIN REDUCTASE (LAR), ANTHOCYANIDIN SYNTHASE (ANS),* and *ANTHOCYANIDIN REDUCTASE (ANR)* (Nesi et al., 2000; Figure 2). The transcript accumulation of *DFR* and *ANR* starts early in seed development, specifically in flowers and young siliques. DFR and ANR are the major enzymes for seed coat coloration in Arabidopsis (Appelhagen et al., 2014; Debeaujon et al., 2003; Devic et al., 1999; Nesi et al., 2000; Nesi, Jond, Debeaujon, Caboche, & Lepiniec, 2001).

Flavonoid biosynthesis is regulated by a combinatorial action of a highly conserved MBW complex consisting of various members of myeloblastosis (MYB), bHLH transcription factor, and WD40 repeat protein (WDR) families (Ramsay & Glover, 2005; Xu, Dubos, & Lepiniec, 2015; Zhang & Schrader, 2017). The WDR protein provides a scaffold allowing protein–protein interactions between the MYB and bHLH proteins, whereas bHLH serves as a docking site for multiprotein interactions (Feller, Hernandez, & Grotewold, 2006). Using the *tt* mutants and rescue experiments, a ternary complex containing TT2 (R2R3 MYB), TT8 (bHLH), and TTG1 (WDR) has been identified that regulates the expression of *DFR* and *ANR* genes, and PA biosynthesis in Arabidopsis seeds (Baudry et al., 2004; Debeaujon et al., 2003; Ramsay & Glover, 2005; Xu et al., 2015). Orthologs of genes involved in this ternary complex have shown to play similar roles and affect seed coat color in many other plant species (Bogs, Jaffé, Takos, Walker, & Robinson, 2007; Carey, Strahle, Selinger, & Chandler, 2004; James et al., 2017; Li et al., 2012, 2016; Liu, Jun, & Dixon, 2014; Lu, Roldan, & Dixon, 2017). Recently, convergent evolution of the *P* gene, an orthologue of *AtTT8*, was demonstrated in different market classes of white‐seeded beans. A multitude of loss‐of‐function alleles were identified as the cause of white seed color in common bean (McClean et al., 2018).

Here, we report that the SD trait and PA accumulation in pinto bean are regulated by the same bHLH transcription factor *P*, meaning that *sd* is another allele at *P* (*P^sd^*). While ectopic expression of *P* from CDC Pintium (RD) completely restores the activity of *DFR* and *ANR* in Arabidopsis *tt8* mutants, and reinstates the wild‐type seed coat phenotype, *P^sd^* from 1533‐15 (SD) only partially rescues the mutant phenotype. Sequence comparison of *P* from pintos that differ in postharvest seed coat color identified trait‐specific sequence polymorphisms which can be used for the development of new gene‐specific molecular markers for breeding.

## 2 MATERIALS AND METHODS

### 2.1 Plant materials and growth conditions

*A. thaliana* ecotype Wassilewskija (Ws‐2) and *tt8* mutant seeds (line: DEBTV122T3) were obtained from Institut Jean‐Pierre Bourgin, Versailles, France. Seeds for pinto bean CDC Pintium and 1533‐15 were developed at the University of Saskatchewan, Canada. Seeds for the cultivars Sundance, StayBright, Croissant (Brick et al., 2011), Centennial, and DR Wood were obtained from Dr. Mark A, Brick, Colorado State University, USA; Lariat and ND‐Palomino were from Dr. Juan M. Osorno, North Dakota State University, USA (Osorno, 2010; Osorno et al., 2018) and Pinto Saltillo (Sanchez‐Valdez, Acosta‐Gallegos, Ibarra‐Pérez, Rosales‐Serna, & Singh, 2004) and SanLuis were obtained from Dr. Jorge Alberto Acosta‐Gallegos, Instituto Nacional de Investigaciones Forestales, Agrícolas y Pecuarias (INIFAP) Celaya, Mexico. Cranberry‐like bean Wit‐rood boontje and RILs obtained from a cross between Wit‐rood boontje and 1533‐13 were obtained from the University of Guelph, Canada. The postharvest seed coat color of the lines used in this study is shown in Table S1. All plants were grown in a growth room under 16 hr light at 25°C and 8 hr dark at 20°C cycle.

Surface‐sterilized pinto bean seeds were grown in Pro‐Mix PGX soil under a light intensity of 300–400 μmol photons m^−2^ s^−1^. *A. thaliana* seeds were surface sterilized and grown on Murashige and Skoog medium for 1 week then transferred to the soil mix and grown at a light intensity of 150 μmol photons m^−2^ s^−1^. *Nicotiana benthamiana* plants were grown in Pro‐Mix PGX soil under the light intensity of 80 μmol photons m^−2^ s^−1^.

### 2.2 Postharvest seed coat darkening

An accelerated seed coat darkening method previously described by Junk‐Knievel et al. (2007) was followed with slight modification. Mature dry bean seeds were placed in open petri dishes directly under a germicidal ultraviolet C (UVC) light bulb (40 watts, λ = 254 nm; model G40T10, Ushio America, Inc.) for 48 hr. The distance between the beans and UVC light bulb was 30 cm. The UVC bulb emitted a light intensity of 2.3 mW/cm^2^.

### 2.3 In silico analysis

Nucleotide and protein sequences, gene location, and tissue‐specific gene expression data were retrieved from the *P. vulgaris* v2.1 database on Phytozome, the Plant Comparative Genomics portal (https://phytozome.jgi.doe.gov/pz/portal.html#!info?alias=Org_Pvulgaris). Primer sequences for SSR markers Pvsd‐1157, Pvsd‐1158, and Pvsd‐0028 from Felicetti et al. (2012) were used in a BLAST search against the *P. vulgaris* whole‐genome database to obtain their exact physical locations. The physical locations of the markers were then used as a reference to find potential candidate *Sd* genes. For phylogenetic analysis, protein sequences were aligned in Clustal Omega, and the tree was constructed by neighbor‐joining method with the bootstrap set to 1,000 using MEGA7 software (Kumar, Stecher, & Tamura, 2016; Sievers et al., 2011). Protein motif and molecular weight predictions were performed using GenomeNet (https://www.genome.jp/tools/motif/) and ExPASy (https://web.expasy.org/compute_pi/), respectively.

### 2.4 RNA extraction, reverse transcription‐PCR, and quantitative RT‐PCR

Total RNA from bean seed coat or seed and Arabidopsis siliques were extracted using the RNeasy Plant Mini kit (Qiagen). On‐column DNA digestion was performed using DNase I (Promega). cDNA was synthesized from total RNA (1.0 μg) using the Platinum™ Quantitative RT‐PCR ThermoScript™ One‐Step System (Invitrogen). Platinum^®^ Taq DNA Polymerase High Fidelity (Invitrogen) was used for PCR, and the amplicons were purified using NucleoSpin^®^ Gel and PCR Clean‐up kit (Takara Bio USA, Inc.).

For qRT‐PCR analysis, cDNA synthesized from RNA isolated from Arabidopsis siliques was used as a template with SsoFast EvaGreen Supermix (BioRad) and gene‐specific primers (Table S2). The expression was normalized to *AtUBQ5* (accession AT3G62250). Each experiment included two biological replicates, with three technical replicates. The data were analyzed using CFX Maestro (Bio‐Rad), and graphs were created using GraphPad Prism 5.0 (www.graphpad.com).

### 2.5 Plasmid construction

The coding regions of *P* or *P^sd^* transcript variants were amplified using RT‐PCR with gene‐specific primers (Table S2) and cloned into pDONR‐Zeo (Invitrogen) using BP clonase (Invitrogen), followed by transformation into *Escherichia coli* DH5α via electroporation. The recombinant entry plasmids were sequence confirmed and recombined using LR clonase (Invitrogen) with two destination vectors, pEarleyGate101 and pMDC32, for subcellular localization and Arabidopsis transformation, respectively. The recombinant destination plasmids were transformed into *Agrobacterium tumefaciens* GV3101.

### 2.6 Subcellular localization and confocal microscopy

For subcellular localization, *A. tumefaciens* harboring pEarlyGate101 containing *P* and *P^sd^* transcript variants from CDC Pintium and 1533‐15, respectively, were infiltrated into leaves of 4‐ to 6‐week‐old *N. benthamiana* plants using the methods of Sparkes, Runions, Kearns, and Hawes (2006). The epidermal cell layers of the infiltrated leaves were visualized 48 hr postinfiltration, using an Olympus FV1000 confocal microscope (Olympus Corporation) with a 60× water immersion objective lens. To validate the localization, *A. tumefaciens* containing nuclear marker translationally fused to cyan fluorescent protein (CFP) was co‐infiltrated at 1:1 ratio with the expression clone. For YFP visualization, the excitation wavelength was set to 514 nm, and emission was collected at 530–560 nm. For CFP visualization, the excitation wavelength was set to 434 nm and emission was collected at 470–500 nm. To visualize the colocalization of the YFP and CFP signals, the ‘Sequential Scan’ tool was used.

### 2.7 Complementation of *Attt8* mutant

Arabidopsis *tt8* plants were transformed with *A. tumefaciens* harboring pMDC32‐P‐1 or pMDC32‐P‐2 from CDC Pintium or pMDC32‐P^sd^‐1 or pMDC32‐P^sd^‐2 from 1533‐15 using the floral dip infiltration method (Clough & Bent, 1998). T1 plants were selected on MS plates supplemented with hygromycin (50 mg/L), and the resistant seedlings were transferred to soil, followed by genotyping to confirm presence of *P* or *P^sd^*. Seeds from all transformants were collected and used for PA analysis.

### 2.8 PA analysis

For qualitative analysis, Arabidopsis wild‐type (Ws‐2), *tt8* mutant, and T2 seeds from all transformants were stained with 0.5% (w/v) 4‐dimethylamino‐cinnamaldehyde (DMACA) for 36 hr in dark followed by de‐staining with 70% ethanol (Li, Tanner, & Larkin, 1996; Zhao & Dixon, 2009). DMACA reacts with PAs/Flavan 3‐ols and produces dark brown color. Photographs were taken using a SMZ1500 dissecting microscope with camera (Nikon).

For quantitative analysis of PA, ground Arabidopsis seeds (0.02–0.03 mg) were extracted with 10 volumes of 50% acetonitrile. Samples were sonicated for 20 min at 4°C followed by centrifugation at 11,200 *g* for 10 min at 4°C. Supernatants were collected, and used for spectrophotometric assay as described by Freixas et al. (2017). Absorbance was measured at 640 nm using Synergy^TM^ 2 Multi‐Detection microplate reader (BioTek). Proanthocyanidin A2 was used as a standard (BOC Sciences).

### 2.9 DNA extraction and high‐resolution melt‐curve qPCR

For genomic DNA extraction, two frozen leaf discs from pinto beans were ground with metal beads in a MM300 Mixer Mill tissue disrupter (Retsch) and DNA was extracted using a conventional cetyl‐trimethylammonium bromide (CTAB) method (Murray & Thompson, 1980). DNA was quantified with a NanoDrop 1000 spectrophotometer (ThermoFisher Scientific). High‐resolution melt‐curve (HRM) analysis was performed to separate the SD and RD lines of pinto bean cultivars using Precision Melt Supermix (Bio‐Rad) with the gene region‐specific primers to detect SNP or 3 bp deletion (InDel; Table S2). The CFX96 Touch Real‐Time PCR Detection System (Bio‐Rad) was precalibrated using the Melt Calibration kit (Bio‐Rad). Samples were run using 95°C for 2 min to activate the hot‐start polymerase, followed by 40 cycles of 95°C for 10 s and 56.5°C for 30 s. For HRM analysis, PCR products were subjected to the following steps: 95°C for 30 s, 60°C for 1 min, and then temperature increase from 65°C to 95°C with a ramp rate of 0.2°C/s. Melt curves were generated and analyzed using Precision Melt Analysis Software 1.3 (Bio‐Rad).

## 3 RESULTS

### 3.1 Comparison of physical and linkage distances identifies a candidate *sd*: *P^sd^*

**FIGURE 3:**
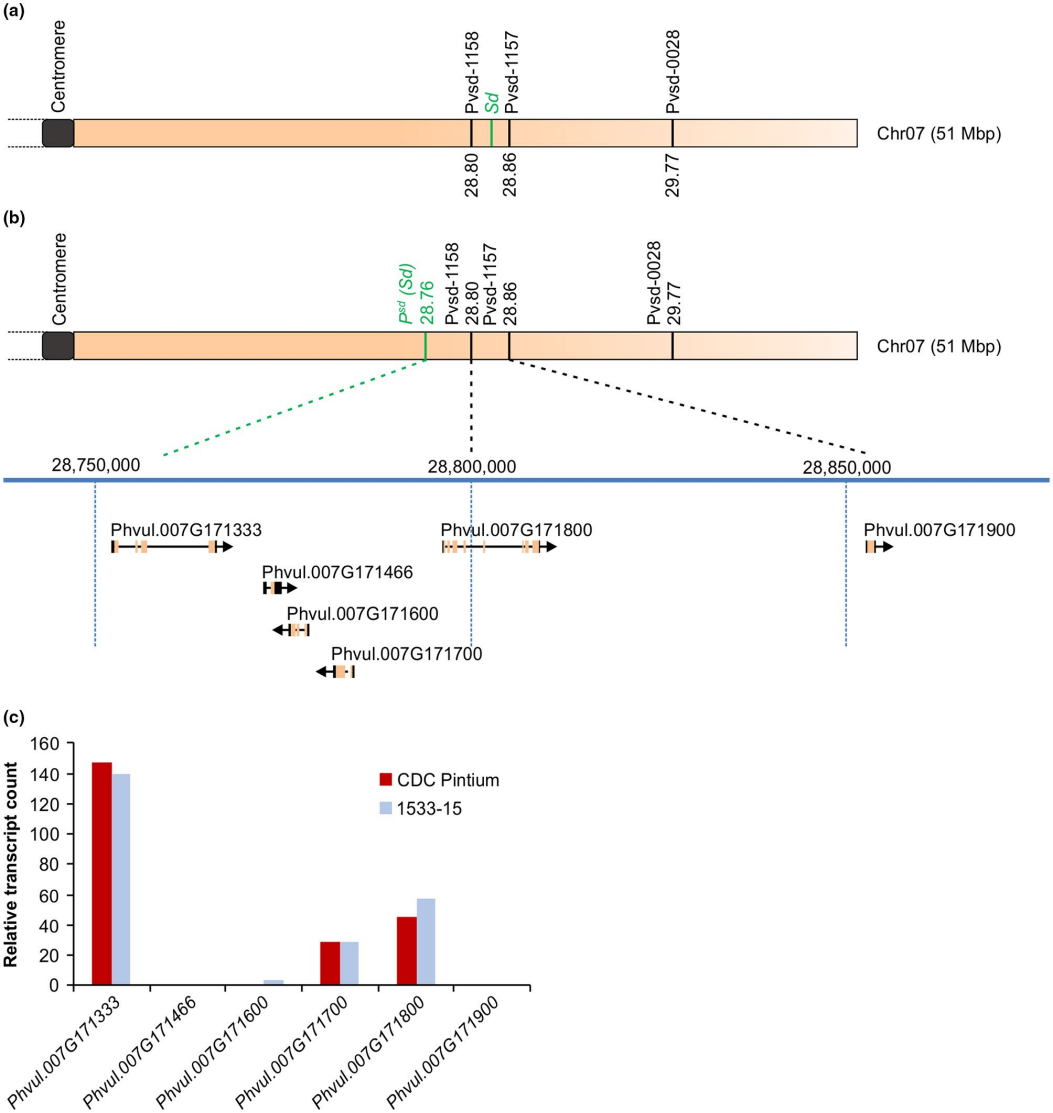
Identification of candidate *Sd* genes located on chromosome 7. (a) Physical locations of SSR markers Pvsd‐1158, Pvsd‐1157, and Pvsd‐0028 are shown. Location of *Sd* as predicted earlier (Felicetti et al., 2012) is indicated. (b) Sequence analysis of 50 kb up‐ and down stream of the tightly linked SSR marker Pvsd1158. (c) RNAseq data from seed coat tissues of CDC Pintium (RD) and 1533‐15 (SD; Duwadi et al., 2018) were mined for the expression analysis of candidate *Sd* genes. Expression is shown in transcript counts

Previously, a genetic analysis mapped *sd* on chromosome 7 tightly linked with the SSR markers, Pvsd‐1157, Pvsd‐1158, and Pvsd‐0028 at 0.9, 0.4, and 3.1 cM, respectively, and placed *sd* between Pvsd‐1157 and Pvsd‐1158 (Felicetti et al., 2012). We identified the location of the markers Pvsd‐1157, Pvsd‐1158, and Pvsd‐0028 on chromosome 7 of the common bean genome assembly v2.1 (Schmutz et al., 2014) using their primer sequences. As shown in Figure 3a, Pvsd‐1158 is located closer to the centromere on the longer arm of chromosome 7, and the physical distance between Pvsd‐1157 and Pvsd‐1158 is 57,713 bp. Another marker, Pvsd‐0028, also reported by Felicetti et al. (2012), is located further downstream from Pvsd‐1157 (Figure 3a). A search for open reading frames (ORFs) between Pvsd‐1158 and Pvsd‐1157 SSR markers uncovered *RAS GTPase* (*Phvul.007G171800*) and *FANTASTIC FOUR* (*FAF*, *Phvul.007G171900*) with the Pvsd‐1158 marker located within *Phvul.007G171800* (Figure 3b). Since gene functions retrieved from the published literature for these two genes did not identify any link with seed coat color, we extended the search to 50 kb upstream and downstream of Pvsd‐1158. This search identified four genes annotated as: *PENTATRICOPEPTIDE REPEAT* (*PPR, Phvul.007G171700*), two uncharacterized genes (*Phvul.007G171466* and *Phvul.007G171600*), and *TRANSPARENT TESTA8* (*TT8, Phvul.007G171333*; Table 1; Figure 3b). Transcript levels of all six candidate genes were determined in the seed coat tissues of CDC Pintium and 1533‐15 using our previously obtained RNAseq data (Duwadi et al., 2018). Based on the functional annotation, *Phvul.007G171333 (P)* was selected as the most appropriate candidate for *Sd* as its expression was highest in seed coat tissue among the six candidates in both pinto cultivars, CDC Pintium and 1533‐15 (Figure 3c), and it had already been implicated in seed coat color of common bean (McClean et al., 2018). Furthermore, the linkage and physical distances between the SSR markers and *Sd* in an F_2_ population (Felicetti et al., 2012) provide additional support for *P* as the *Sd* gene. Based on our analysis, *Sd* resides closer to the SSR marker Pvsd‐1158 (the tightly linked marker), and Pvsd‐1158 is located between *Sd* and Pvsd‐1157 as shown in Figure 3b. These findings suggest that *sd* could be a novel allele of the bean *P* gene and would be renamed *P^sd^*.

**TABLE 1:** List of genes present within 50 kb of Pvsd‐1158 (Felicetti et al., 2012) in pinto bean

Gene	Distance from Pvsd‐1158 (bp)	Database annotation
*Phvul.007G171333*	36,576	Transcription factor TT8
*Phvul.007G171466*	27,988	Unknown
*Phvul.007G171600*	24,429	Unknown
*Phvul.007G171700*	18,330	PPR repeat
*Phvul.007G171800*	0	Ras homolog gene family (RAS)
*Phvul.007G171900*	49,801^a^	Fantastic four meristem regulator (FAF)

^a^ Position of gene downstream of the marker.

### 3.2 Sequence analysis and characterization of *P^sd^*

*P* contains a 1,992 bp open reading frame (ORF) that is predicted to encode a bHLH transcription factor with a calculated molecular mass of 73.8 kDa and pI of 5.15 while *P^sd^* ORF is of 1,995 bp. Since it was annotated as TT8, we performed a multiple sequence alignment with characterized TT8 from other plant species. Similar to other bHLH proteins and previously reported P (McClean et al., 2018), P^sd^ contains all four motifs: an N‐terminal MYB interaction region (MIR), followed by an activation domain (AD), a signature bHLH, and an ACT (aspartate kinase, chorismate mutase, and TyrA) domain at the C‐terminus that are found in other characterized TT8 proteins (Figure S1). Unlike other *TT8*s, *P^sd^* has an extra exon at the 3’ end. Exon 7 and exon 8 of *P^sd^* are separated by a small intron (92 bp). Phylogenetic analysis of P^sd^ with other bHLH proteins with seed coat color‐related function showed that P^sd^ is in the same clade as other legume TT8 proteins (Figure S2b).

**FIGURE 4:**
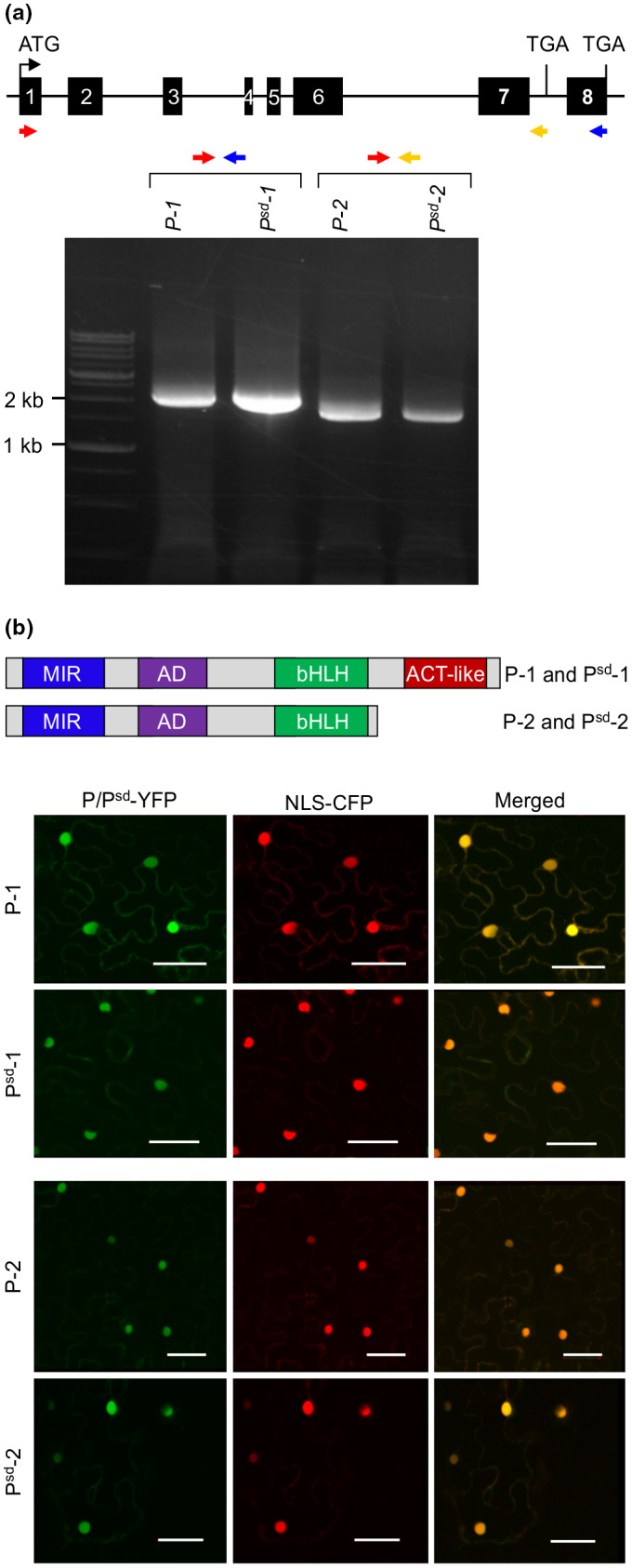
Characterization of *P^sd^*. (a) *P* gene model. Boxes indicate exons and lines indicate introns. The gel picture shows two transcript variants *P‐1* and *P‐2* in CDC Pintium (RD) and *P^sd^‐1* and *P^sd^‐2* in 1533‐15 (SD). Primer locations for amplifying the protein coding region of *P* and *P^sd^* using RT‐PCR are indicated. (b) Predicted protein motifs of P‐1, P^sd^‐1, P‐2, and P^sd^‐2 include MYB interaction region (MIR), activation domain (AD), bHLH domain, and ACT‐like motif (only present in P‐1 and P^sd^‐1). *P‐1* and *P‐2* from CDC Pintium and *P^sd^‐1*, and *P^sd^‐2* from 1533‐15 were translationally fused upstream of YFP reporter gene, cotransformed with nuclear localization signal fused cyan fluorescent protein (NLS‐CFP) into *Nicotiana benthamiana* via *Agrobacterium tumefaciens*, and visualized in leaf epithelial cells by confocal microscopy. Merged signal was collected by sequential scanning of YFP and CFP channels. Scale bars = 40 µm

To determine if there is any sequence variation between RD and SD pinto lines, transcript sequences of this gene from CDC Pintium and 1533‐15 were analyzed. Primers were designed using the reference gene sequence to amplify the full protein coding region from both, and RT‐PCR was performed. The amplicons were cloned into pGEM^®^‐T vector and sequenced. The comparison of coding DNA sequences of *P* from CDC Pintium and *P^sd^* from 1533‐15 identified three single nucleotide polymorphisms at positions 316 (T to C), 1,334 (G to A), and 1,406 (G to A) leading to the changes in the amino acids F106L, A445T, and R468H. Furthermore, a 3‐nucleotide insertion was detected at position 751 (4,045 genomic DNA) in 1533‐15. This insertion leads to the gain of a glutamate (E) residue that lies in the activation domain of the bHLH protein (Figure S3b). The sequence analysis also revealed that in addition to the predicted size transcript, there is a transcript variant with a 1,658 bp ORF. The transcript variant contains an intron (intron 7) that introduces a premature stop codon (Figure 4a). This was confirmed by RT‐PCR using gene‐specific primers where the reverse primer was located in intron 7 (Figure 4a). Two transcripts were found for *P* or *P^sd^* in all RD and SD cultivars used in this study. As shown in Figure S4, RD cultivars contained *P‐1* (1,992 bp) and *P‐2* (1,658 bp) transcripts and SD cultivars contained *P^sd^‐1* (1,995 bp) and *P^sd^‐2* (1,661 bp). The predicted P‐2 and P^sd^‐2 proteins lack the ACT‐like motif which is present in P‐1, P^sd^‐1, and other TT8 orthologs (Figure 4b).

As a predicted transcription factor, P is expected to be present in the nucleus. To evaluate if the sequence variation within *P* and *P^sd^* and their transcript variants in the two pinto cultivars result in differential localization in the subcellular compartment, we translationally fused both transcript variants from CDC Pintium (RD) and 1,533‐15 (SD) with YFP, and transiently expressed them in *N. benthamiana* leaves for subcellular localization. The results revealed that the lack of ACT domain does not alter the subcellular localization, and that the two variant proteins from both cultivars localize in the nucleus (Figure 4b).

### 3.3 P^sd^ regulates PA biosynthetic genes and PA content in seed coat

**FIGURE 5:**
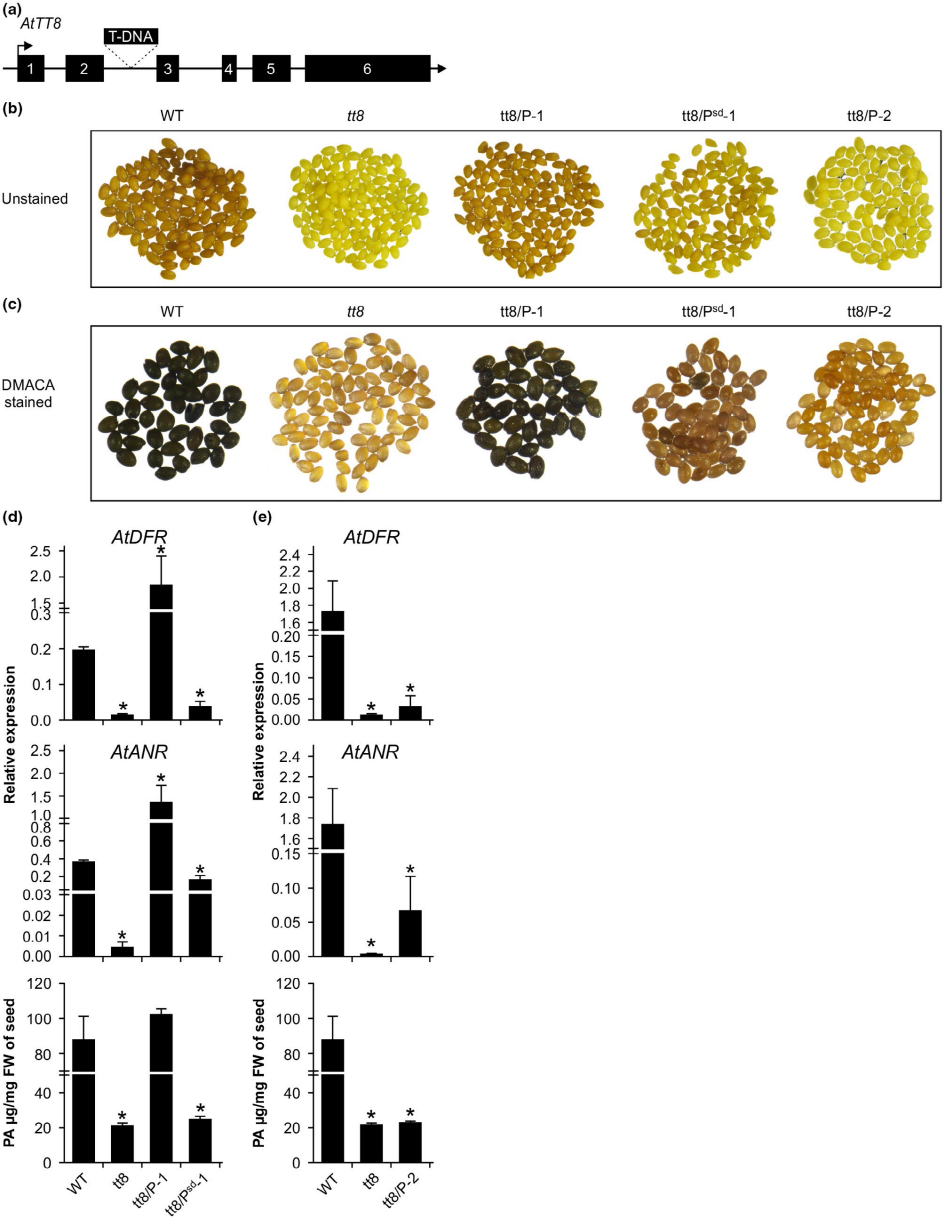
Functional analysis of *P^sd^*. (a) Arabidopsis *TT8* gene structure showing the T‐DNA insertion in the second intron. Introns are represented by a line and exons are represented by a rectangle with exon number. (b) Genetic complementation of *tt8* in Arabidopsis by variants of *P*. Seed coat phenotype of wild‐type (WT), *tt8*, and T2 complementation lines overexpressing *P‐1* and *P‐2* from CDC Pintium and *P^sd^‐1* from 1533‐15 are shown. (c) DMACA stained versions of same seeds as in b). Transcript and PA accumulation in (d) tt8/P‐1 and tt8/P^sd^‐1 and (e) tt8/P‐2 complementation lines. Expression analysis of *AtDFR* and *AtANR* using RT‐qPCR analysis was performed on siliques collected from T1 plants. T2 seeds were used measuring PA content. Total extractable proanthocyanidin levels are expressed as proanthocyanidin A2 equivalents as described under ‘Section 2’. All data represent the mean ± *SEM* of at least two biological and three technical replicates. Asterisks (*) represent significant difference compared to WT as determined by Student's *t* test (*p* < .05)

Arabidopsis TT8 is a bHLH transcription factor that regulates PA biosynthetic genes, and influences seed coat pigment biosynthesis (Nesi et al., 2000). Seeds of non‐functional AtTT8 lack brown pigment and appear pale yellow. Since bean transformation is extremely difficult, for the functional characterization of P^sd^ we sought to demonstrate that it is a true functional ortholog of AtTT8. For this, we complemented Arabidopsis *tt8* by genetic transformation with constructs harboring *P‐1* or *P‐2* from CDC Pintium and *P^sd^‐1* from 1533‐15, and analyzed the T2 seeds collected from multiple independent T1 transgenic lines. Analysis of seed coat phenotype of T2 seeds revealed that all nine tt8/P‐1*‐*CDC Pintium transgenic lines completely restored wild‐type phenotype, and all 26 tt8/P^sd^‐1‐1533‐15 lines only partially restored the wild‐type phenotype. None of the 11 tt8/P‐2*‐*CDC Pintium lines rescued the mutant phenotype suggesting that truncated P‐2 is either non‐functional or has acquired some alternate function. Figure 5b,c show the representative photographs of unstained and DMACA‐stained seeds of wild‐type, *tt8* mutant, and tt8/P‐1*‐*CDC Pintium, tt8/P^sd^‐1‐1533‐15, and tt8/P‐2*‐*CDC Pintium lines. Four independent transformed lines representing CDC Pintium or 1533‐15 were further investigated in detail for PA level and PA biosynthetic gene expression (Figure S5).

To elucidate how *P‐1* and *P^sd^‐1* rescue the *tt8* phenotype, we investigated the expression of *DFR* and *ANR* in the tt8/P^sd^ and tt8/P lines. Young siliques were collected from wild‐type, *tt8* and T1 complementation lines, and RT‐PCR was performed to confirm their status. The presence of *AtTT8* only in the wild‐type but not in other samples verified the mutant background, and the presence of *P^sd^* or *P* only in transformed lines, confirmed that the transformations were accomplished successfully (Figure S6a,b). The transcript levels of *DFR* and *ANR* genes were measured by quantitative RT‐PCR using gene‐specific primers in multiple independent lines transformed with the gene from either CDC Pintium or 1533‐15. The results revealed that *DFR* and *ANR* transcripts were present in wild‐type but almost absent in *tt8* (Figure 5d). Overexpression of *P‐1* in *tt8* dramatically increased the accumulation of *DFR* and *ANR* transcripts to 7.25‐ and 4.2‐fold, respectively, in *tt8*/P‐1‐CDC Pintium plants compared to the wild‐type. No such increase in transcript accumulation was observed for tt8/P^sd‐1^‐1533‐15 plants when compared to wild‐type. Even though an increase in *ANR* transcript was detected in tt8/P^sd^‐1‐1533‐15 lines compared to mutants, the *DFR* transcript levels were similar to that of *tt8* lines. Additionally, *P^sd^‐1* transcript accumulation was higher in tt8/P^sd^‐1‐1533‐15 lines than *P‐1* in tt8/P‐1‐CDC Pintium lines (Figure S6b). Analysis of PA content in the complementation lines showed that tt8/P‐1‐CDC Pintium lines contained the same level of PAs as wild‐type while it was significantly lower in tt8/P^sd^‐1‐1533‐15 lines (Figure 5d). These results suggest that lack of DFR synthesis is a bottleneck for PA biosynthesis, and that *P‐1* and *P^sd^‐1* sequence variation between CDC Pintium and 1533‐15 reduces expression level in 1533‐15. tt8/P^sd^‐2‐CDC Pintium plants did not express *DFR* and *ANR* to the same level as wild‐type plants thereby showing the decreased levels of PA accumulation as the mutant (Figure 5e; Figure S7d,e) demonstrating the importance of the ACT motif in the P protein.

### 3.4 Two sequence‐specific polymorphisms define SD trait in pintos

**FIGURE 6:**
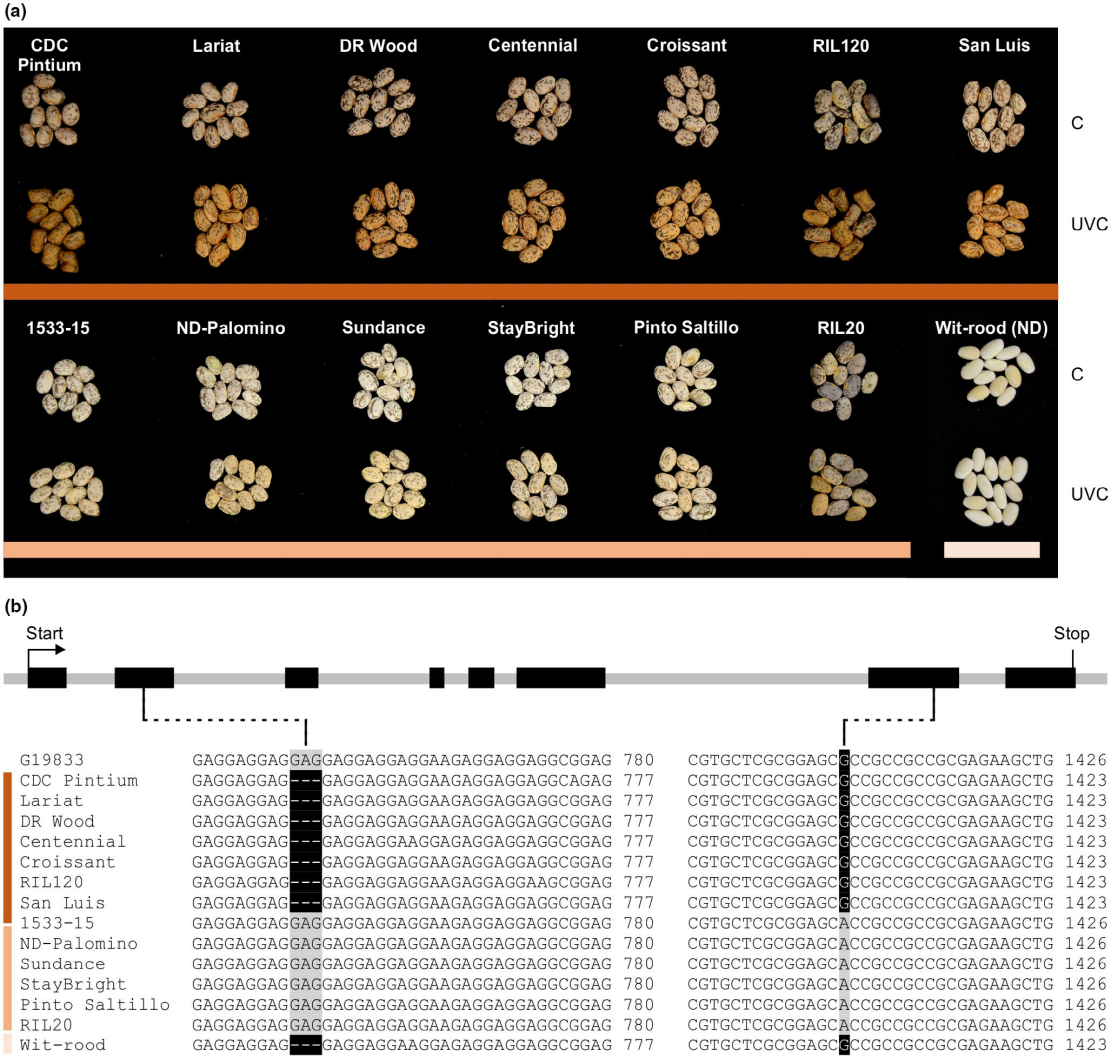
RD‐ and SD‐specific polymorphisms in pinto bean lines. (a) Photograph of RD and SD lines before and after UVC light treatment to correlate sequence polymorphisms with postharvest seed coat darkening effect. An ND type cranberry‐like bean Wit‐rood boontje was also included as a control. RIL 120 (RD) and RIL 20 (SD) represent RILs obtained from the Wit‐rood boontje and 1533‐15 cross. C, untreated and UVC, treated. (b) Genotypes of SD, RD, and ND pinto bean lines and two representative SD and RD RILS from Wit‐rood boonjie and 1533‐15 cross showing a 3 nucleotide InDel (GAG) in exon 2 and a SNP (G to A) in exon 7 of *P* that associate with differences in darkening phenotype

To investigate if any of the sequence polymorphisms within *P* between CDC Pintium and 1533‐15 are present in other pinto lines, and if those polymorphisms could be used for the development of gene‐specific markers for the SD trait that could help pinto bean breeders with regards to the postharvest seed coat darkening issue, we compared transcript sequences among 21 pinto bean lines that show differential postharvest seed coat darkening phenotype. Of 21 lines, nine were recombinant inbred lines (RILs) developed from a Wit‐rood boontje (ND) × 1533‐15 (SD) cross (Erfatpour, Navabi, & Pauls, 2018). The postharvest seed coat darkening trait in these cultivars was confirmed by exposing the seeds to UVC light and observing the relative change in seed coat color. Based on the seed coat color, the pintos grouped into SD, RD, or ND (Figure 6a). The sequence analysis of all 21 lines consistently detected 1 SNP (G/A) and a 3 nucleotide insertion (GAG) in SD beans as compared to RD (Figure 6b). The ND bean (Wit‐rood boontje) included in this study grouped with RD pintos in terms of these polymorphisms. Our results confirm that the two sequence polymorphisms (SNP and InDel) identified here correlate with the slow darkening (SD) postharvest seed coat trait.

### 3.5 Epistatic inheritance of *J* and *Sd* confirms *P^sd^* is *Sd*

Two unlinked recessive alleles of *J* and *Sd* are responsible for the ND and SD seed coat phenotypes, respectively (Elsadr et al., 2011). Analysis of the extent of postharvest seed coat darkening in the RILs derived from Wit‐rood boontje (ND) and 1533‐15 (SD) parents (Erfatpour et al., 2018) uncovered the presence of RD trait in some RILs, although neither parent was RD. Our analysis confirmed that the *P* gene sequence in Wit‐rood boontje matches with that of the RD lines (Figure 6b). Therefore, the RILs with RD phenotype must have inherited the *P* allele from Wit‐rood boontje (Figure S7). This supports the inheritance study of seed coat postharvest darkening explained previously by Elsadr et al. (2011), and provides further evidence that the SD phenotype is controlled by *P^sd^*.

### 3.6 Development of gene‐specific markers for the SD trait

**FIGURE 7:**
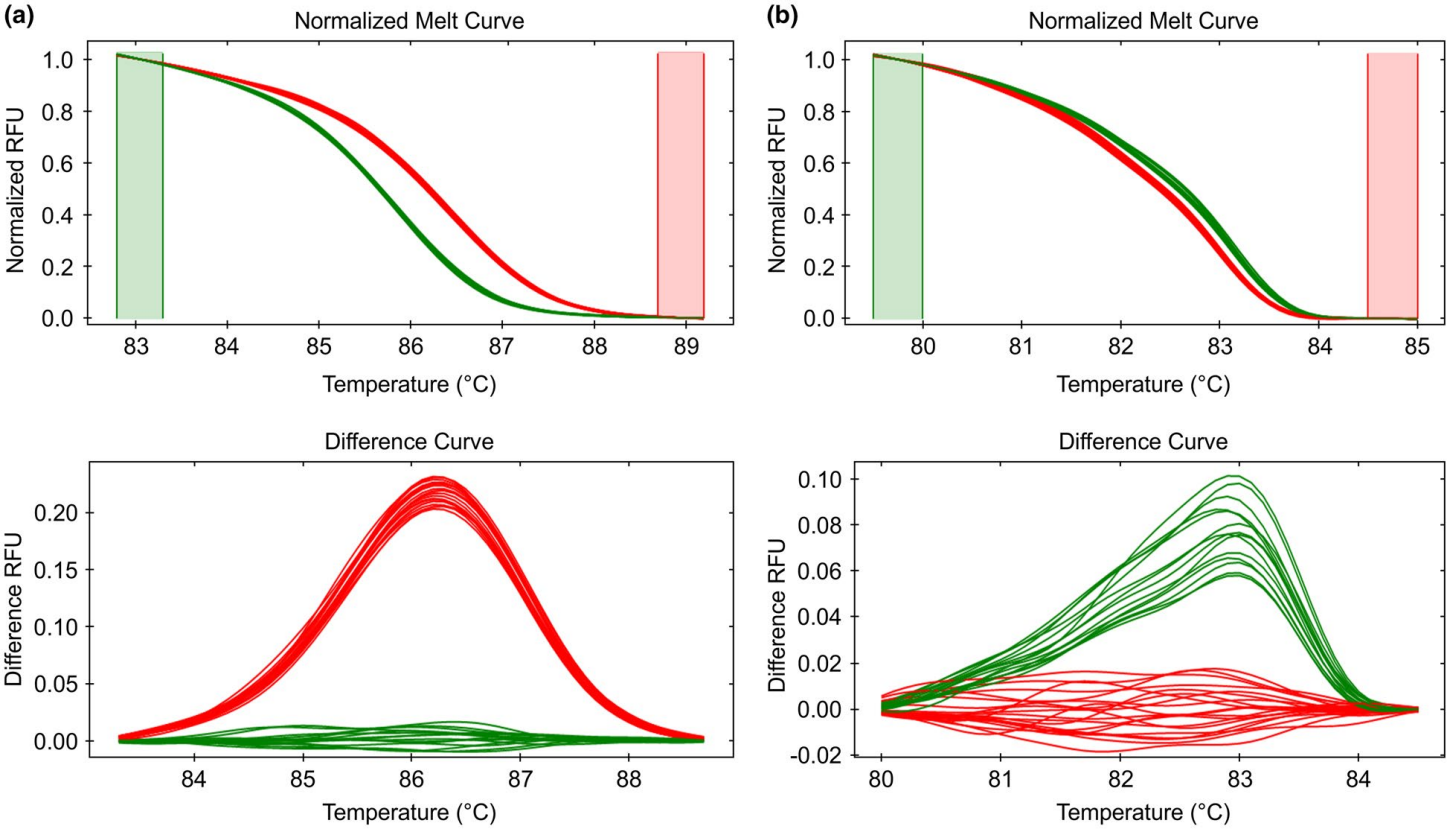
High‐resolution melt‐curve quantitative PCR (HRM‐qPCR) analysis of RD and SD type pinto beans with (a) SNP (PvsdSNP) and (b) InDel (PvsdInDel) markers. Assays differentiate RD (red) and SD (green) pinto lines. Normalized and difference curve for relative fluorescence unit (RFU) with temperature change is shown

To develop markers that can distinguish RD from SD lines, primers were designed to cover the SNP or the InDel region, and a high‐resolution melting analysis was performed on genomic DNA isolated from all 21 pinto lines to detect the polymorphisms. The SNP marker (PvsdSNP) spans 75 nucleotides across the G/A polymorphism and provides a tight difference curve separating SD and RD pinto into two different groups (Figure 7a). The InDel marker (PvsdInDel) covered a 125 nucleotide region spanning the InDel. As some cultivars contained non‐specific/nonsense mutations within the amplified region, a tighter difference curve for the InDel marker could not be obtained (Figure 7b). Nevertheless, PvsdInDel was able to differentiate SD and RD genotypes, and could be used as a marker for SD trait.

## 4 DISCUSSION

Here, we demonstrate that another allele at the common bean *P* gene *P^sd^* is responsible for the SD trait in pinto bean. *P* is a bHLH transcription factor that restores seed coat color in Arabidopsis *tt8* mutants by regulating the late PA biosynthetic genes and PA levels. Sequence comparison of this gene in several pintos that differ in postharvest seed coat darkening provided insights into the molecular mechanism governing this trait and development of new gene‐specific markers that could be utilized in bean breeding programs.

Previously, *Sd* was shown to be located between the SSR markers Pvsd‐1158 and Pvsd‐1157 with a tighter linkage to Pvsd‐1158 in both RIL and F2 populations (Felicetti et al., 2012). A search for genes in the linkage region of Pvsd‐1158 on chromosome 7 identified six protein coding loci and repositioned *Sd* with respect to the previously identified SSR markers (Figure 3b). The ORFs, *Phvul.007G171800* and *Phvul.007G171900*, potentially encoding for *RAS* and *FAF* that are located within or in between the SSR markers Pvsd‐1158 and Pvsd‐1157, do not possess previously known seed coat or pigment‐related function, and their transcript levels were low in bean seed coat (Figure 3c; Table 1). In Arabidopsis, *FAF* expresses in the centre of the shoot meristem, and functions in organogenesis (Wahl, Brand, Guo, & Schmid, 2010). RASs are small GTPases that act as molecular switches in linking extracellular signals to intracellular networks that impact a wide variety of cellular processes in eukaryotes. However, as Arabidopsis does not contain any RAS homolog (Vernoud, Horton, Yang, & Nielsen, 2003), *P. vulgaris RAS* gene may not be related to seed coat color. Among the four other loci that are linked with the marker Pvsd‐1158 are two genes with unknown function, as well as a PPR protein encoding gene and *P*, a bHLH transcription factor already associated with seed coat color (McClean et al., 2018). The transcript abundance of *P* in seed coat, its location near the most tightly linked marker, and putative function as TT8 suggested that a new allele at *P* is the *Sd* gene (Figure 3,). *TT8* regulates seed PA biosynthesis in Arabidopsis as does its homologue in *Medicago truncatula*, and mutation in this gene results in reduced seed pigmentation and PA accumulation (Li et al., 2016; Nesi et al., 2000). The fact that CDC Pintium (RD) accumulates significantly higher levels of PAs and PA monomers such as catechin compared to 1533‐15 (SD) in immature seed coat and mature seeds (both aged and non‐aged; Beninger et al., 2005; Duwadi et al., 2018) provided additional support for *P^sd^* as the *Sd* gene, and its function in PA accumulation.

Complementation of the *Attt8* mutant phenotype with *P* and *P^sd^* and restoration of PA levels in tt8/P and tt8/P^sd^ complementation lines demonstrated its function and confirmed that *P* is an ortholog of *AtTT8* in pintos (Figure 5). Of the two transcript variants of *P* and *P^sd^*, only *P‐1* and *P^sd^‐1* were able to rescue the *tt8* mutant phenotype. P‐2 and P^sd^‐2 lack the ACT domain that is present in P‐2, P^sd^‐1, and TT8 orthologs from other plant species (Figure 4b; Figure S1). Dimerizations of bHLH‐ or the ACT domain of maize bHLH transcription factor R were shown to act as molecular switches that define gene expression patterns (Kong et al., 2012). When the ACT domain homodimerized, the bHLH domain remained monomeric and did not bind DNA directly but interacted with the C1 (MYB) and R‐interacting factor (RIF1) to activate expression of *A1* gene (*DFR* ortholog) in maize. In the absence of ACT dimerization, the bHLH region of the R protein formed a homodimer, and resumed its DNA binding activity leading to the activation of anthocyanin genes such as *Bz1* which altered the maize aleurone color from purple to red‐brown (Roth, Goff, Klein, & Fromm, 1991; Taylor & Briggs, 1990). Our results demonstrate that the ACT domain in P/P^sd^ is also necessary for its function (Figure 5). Additionally, intron retention in the second transcript also leads to the addition of an additional 19 amino acid residues before premature termination (Figure S3a). Although both protein products resulting from both transcript variants of *P* and *P^sd^* localize to the nucleus as expected for a transcription factor, the absence of the ACT domain and/or additional amino acids in P‐2 and P^sd^‐2 possibly contribute toward its altered function.

TT8 regulates the late biosynthetic genes in the PA pathway (Albert, Delseny, & Devic, 1997; Li et al., 2016; Nesi et al., 2000). Transcript levels of two PA biosynthetic genes, *DFR* and *ANR*, were reduced in *Attt8* as compared to the wild‐type, which affected PA accumulation and seed coat color (Figure 5). Overexpression of *P‐1* from CDC Pintium rescued the *tt8* mutant phenotype by activating the expression of *AtDFR* and *AtANR* genes. Despite significantly higher levels of *AtDFR* (7.2 fold) and *AtANR* (4.2 fold) transcripts in tt8/P‐1‐CDC Pintium plants as compared with the Arabidopsis wild‐type, no increased PA level was observed in these lines. This could be due to the limited substrate pool channeled toward PA biosynthesis in the tt8/P‐1‐CDC Pintium lines. In contrast, tt8/P^sd^‐1‐1533‐15 plants failed to accumulate *AtDFR* and *AtANR* transcripts to the same levels as the wild‐type, and the *tt8* mutant phenotype was not completely rescued. DFR catalyzes the NADPH‐dependent reduction in dihydroflavonols such as dihydroquercetin and dihydrokaempferol to produce flavan 3, 4‐diols, which are intermediates for the biosynthesis of anthocyanin and PA (Figure 2). ANR (also called BAN) reduces cyanidin to epicatechin, one of the units of PA polymers (Devic et al., 1999; Pang, Peel, Sharma, Tang, & Dixon, 2008). We speculate that the R468H substitution in the bHLH domain in 1533‐15 contributes to the reduced activity of P^sd^‐1. The bHLH domain is critical for binding to the promoters of target genes (Heim et al., 2003). The pK_a_ values of the arginine and histidine side chains differ significantly. This change in pK_a_ values possibly affects the binding of PvTT8.1‐1533‐15 to E‐box (CANNTG) or G‐box (CACGTG) motifs of promoters (Atchley & Fitch, 1997; Onufriev & Alexov, 2013). Furthermore, an additional glutamate residue (due to the 3 nucleotide insertion in SD) was consistently observed in the activation domain in six SD type pintos and the bean reference genome line G19833. It is yet to be determined if this InDel is related to the darkening or simply associated sequence wise with the allele. Previously, variations in *P* gene was associated with mottling in beans (Emerson, 1909). Recently, the *P* gene was reported to control seed color and weight in common bean market classes (McClean et al., 2018). Multiple SNPs, deletions, substitutions, and frame‐shift mutations within *P* are depicted in many different white bean market classes leading to a non‐functional bHLH. *P^sd^* is yet another allele of *P*. Recently, a KASP‐based SNP marker within *P* was shown to be tightly linked with SD in carioca and pinto beans (Alvares et al., 2019). Our SNP marker targets the same SNP using a different marker technology. Many genes such as *P*, *C*, *R*, *J*, *D*, *G*, *B*, *V,* and *Rk* control seed coat color in common bean where their epistatic interaction with other genes defines the extent of color and pattern in the market classes (McClean, Lee, Otto, Gepts, & Bassett, 2002). A combinatorial interaction among bHLH (AtTT8), R2R3 MYB (AtTT2), and WDR (AtTTG1) proteins has been shown to regulate late PA biosynthetic genes and PA accumulation in Arabidopsis seeds (Debeaujon et al., 2003; Nesi et al., 2000, 2001; Walker et al., 1999). Mutation in any of the partners involved in this ternary complex reduces or abolishes their protein activity and affects PA accumulation and seed coat color. Based on our results and those of McClean et al. (2018), a reduction in bHLH activity of *P^sd^* in 1533‐15 results in SD phenotype while a non‐functional bHLH encoded by *p* leads to colorless beans.

Studies using crosses among RD, SD, and ND pinto genotypes have confirmed that the inheritance of postharvest seed coat darkening is controlled by two unlinked major genes *J* and *Sd* with recessive epistatic interaction (Elsadr et al., 2011). *J* determines if the seed coat darkens or not and yields a ND phenotype irrespective of the *Sd* genotype. *Sd* determines how quickly a seed coat darkens where *sdsd* genotype darkens slower than *SdSd* or *Sdsd* (Bassett, Lee, Symanietz, & McClean, 2002; Elsadr et al., 2011; McClean et al., 2002). In the ND Wit‐rood boontje, the *P* sequence matches that of RD type pintos (Figure 6b). Therefore, we infer that RD RILs derived from the Wit‐rood boontje x 1533‐15 cross (Figure 7b) inherited *P* from Wit‐rood boontje, and that *J* may encode one of the other proteins in the MBW complex. Further identification of the P partner proteins in the MBW complex may facilitate understanding of the molecular mechanisms involved in the postharvest seed coat darkening process.

## AUTHOR CONTRIBUTIONS

N.S.I. contributed to experimental design, performed all the experiments, collected and analyzed data, and prepared draft manuscript, K.E.B. and K.P.P. provided the germplasm and contributed to manuscript preparation, F.M. contributed to experimental design and manuscript preparation, and S.D. conceived and designed the experiments, supervised all aspects of the project, and prepared final draft manuscript.

## Supporting information

Supplementary Material

## References

[ppp310132-bib-0001] Albert, S. , Delseny, A. , & Devic, M. (1997). *BANYULS*, a novel negative regulator of flavonoid biosynthesis in the *Arabidopsis* seed coat. The Plant Journal, 11(2), 289–299. 10.1046/j.1365-313X.1997.11020289.x 9076994

[ppp310132-bib-0002] Alvares, R. C. , Stonehouse, R. , Souza, T. L. P. O. , Melo, P. G. S. , Miklas, P. N. , Bett, K. E. , … Pereira, H. S. J. E. (2019). Generation and validation of genetic markers for the selection of carioca dry bean genotypes with the slow‐darkening seed coat trait. Euphytica, 215(8), 141. 10.1007/s10681-019-2461-y

[ppp310132-bib-0003] Appelhagen, I. , Thiedig, K. , Nordholt, N. , Schmidt, N. , Huep, G. , Sagasser, M. , & Weisshaar, B. (2014). Update on *transparent testa* mutants from *Arabidopsis thaliana* *:* Characterisation of new alleles from an isogenic collection. Planta, 240, 955–970. 10.1007/s00425-014-2088-0 24903359

[ppp310132-bib-0004] Atchley, W. R. , & Fitch, W. M. (1997). A natural classification of the basic Helix‐Loop‐Helix class of transcription factors. Proceedings of the National Academy of Sciences of the United States of America, 94(10), 5172–5176. 10.1073/pnas.94.10.5172 9144210 PMC24651

[ppp310132-bib-0005] Bassett, M. J. , Lee, R. , Symanietz, T. , & McClean, P. E. (2002). Inheritance of reverse margo seed coat pattern and allelism between the genes *J* for seed coat color and *L* for partly colored seed coat pattern in common bean. Journal of the American Society for Horticultural Science, 127(1), 56–61. 10.21273/JASHS.127.1.56

[ppp310132-bib-0006] Baudry, A. , Heim, M. A. , Dubreucq, B. , Caboche, M. , Weisshaar, B. , & Lepiniec, L. (2004). TT2, TT8, and TTG1 synergistically specify the expression of *BANYULS* and proanthocyanidin biosynthesis in *Arabidopsis thaliana* . The Plant Journal, 39(3), 366–380. 15255866 10.1111/j.1365-313X.2004.02138.x

[ppp310132-bib-0007] Beninger, C. W. , Gu, L. , Prior, D. C. , Junk, A. , Vandenberg, A. , & Bett, K. E. (2005). Changes in polyphenols of the seed coat during the after‐darkening process in pinto beans (*Phaseolus vulgaris* L.). Journal of Agriculture and Food Chemistry, 53(20), 7777–7782. 10.1021/jf050051l 16190630

[ppp310132-bib-0008] Bogs, J. , Jaffé, F. W. , Takos, A. M. , Walker, A. R. , & Robinson, S. P. (2007). The grapevine transcription factor VvMYBPA1 regulates proanthocyanidin synthesis during fruit development. Plant Physiology, 143(3), 1347–1361. 10.1104/pp.106.093203 17208963 PMC1820911

[ppp310132-bib-0009] Brick, M. , Ogg, J. , Schwartz, H. , Johnson, J. , Judson, F. , Singh, S. , … Pastor‐Corrales, M. (2011). Registration of ‘Croissant’ pinto bean. Journal of Plant Registrations, 5(3), 299–303. 10.3198/jpr2010.07.0443crc

[ppp310132-bib-0010] Carey, C. C. , Strahle, J. T. , Selinger, D. A. , & Chandler, V. L. (2004). Mutations in the *pale aleurone color1* regulatory gene of the *Zea mays* anthocyanin pathway have distinct phenotypes relative to the functionally similar *TRANSPARENT TESTA GLABRA1* gene in *Arabidopsis thaliana* . The Plant Cell, 16(2), 450–464. 14742877 10.1105/tpc.018796 PMC341916

[ppp310132-bib-0011] Clough, J. S. , & Bent, F. A. (1998). Floral dip: A simplified method for *Agrobacterium*‐mediated transformation of *Arabidopsis thaliana* . The Plant Journal, 16(6), 735–743. 10.1046/j.1365-313x.1998.00343.x 10069079

[ppp310132-bib-0012] Debeaujon, I. , Nesi, N. , Perez, P. , Devic, M. , Grandjean, O. , Caboche, M. , & Lepiniec, L. (2003). Proanthocyanidin‐accumulating cells in *Arabidopsis* testa: Regulation of differentiation and role in seed development. The Plant Cell, 15(11), 2514–2531. 10.1105/tpc.014043 14555692 PMC280558

[ppp310132-bib-0013] Devic, M. , Guilleminot, J. , Debeaujon, I. , Bechtold, N. , Bensaude, E. , Koornneef, M. , … Delseny, M. (1999). The *BANYULS* gene encodes a DFR‐like protein and is a marker of early seed coat development. The Plant Journal, 19(4), 387–398. 10.1046/j.1365-313X.1999.00529.x 10504561

[ppp310132-bib-0014] Dixon, R. A. , Xie, D. Y. , & Sharma, S. B. (2005). Proanthocyanidins – A final frontier in flavonoid research? New Phytologist, 165(1), 9–28. 10.1111/j.1469-8137.2004.01217.x 15720617

[ppp310132-bib-0015] Duwadi, K. , Austin, R. S. , Mainali, H. R. , Bett, K. , Marsolais, F. , & Dhaubhadel, S. (2018). Slow darkening of pinto bean seed coat is associated with significant metabolite and transcript differences related to proanthocyanidin biosynthesis. BMC Genomics, 19, 260. 10.1186/s12864-018-4550-z 29661146 PMC5903001

[ppp310132-bib-0016] Elsadr, H. T. , Marles, M. A. S. , Caldas, G. V. , Blair, M. W. , & Bett, K. E. (2015). Condensed tannin accumulation during seed coat development in five common bean genotypes. Crop Science, 55(6), 2826–2832. 10.2135/cropsci2015.01.0051

[ppp310132-bib-0017] Elsadr, H. T. , Wright, L. C. , Pauls, K. P. , & Bett, K. E. (2011). Characterization of seed coat post harvest darkening in common bean (*Phaseolus vulgaris* L.). Theoretical and Applied Genetics, 123(8), 1467–1472. 10.1007/s00122-011-1683-8 21863347

[ppp310132-bib-0018] Emerson, R. A. (1909). Factors for mottling in beans. Journal of Heredity, 5(1), 368–375. 10.1093/jhered/os-5.1.368

[ppp310132-bib-0019] Erfatpour, M. , Navabi, A. , & Pauls, K. P. (2018). Mapping the non‐darkening trait from ‘Wit‐rood boontje’ in bean (*Phaseolus vulgaris*). Theoretical and Applied Genetics, 131, 1331–1343. 10.1007/s00122-018-3081-y 29502138

[ppp310132-bib-0020] Felicetti, E. , Song, Q. , Jia, G. , Cregan, P. , Bett, K. E. , & Miklas, P. N. (2012). Simple sequence repeats linked with slow darkening trait in pinto bean discovered by single nucleotide polymorphism assay and whole genome sequencing. Crop Science, 52(4), 1600–1608. 10.2135/cropsci2011.12.0655

[ppp310132-bib-0021] Feller, A. , Hernandez, J. M. , & Grotewold, E. (2006). An ACT‐like domain participates in the dimerization of several plant basic‐Helix‐Loop‐Helix transcription factors. Journal of Biological Chemistry, 281(39), 28964–28974. 10.1074/jbc.M603262200 16867983

[ppp310132-bib-0022] Freixas, C. J. A. , Munholland, S. , Silva, A. , Subedi, S. , Lukens, L. , Crosby, W. L. , … Bozzo, G. G. (2017). Proanthocyanidin accumulation and transcriptional responses in the seed coat of cranberry beans (*Phaseolus vulgaris* L.) with different susceptibility to postharvest darkening. BMC Plant Biology, 17, 89. 10.1186/s12870-017-1037-z 28545577 PMC5445279

[ppp310132-bib-0023] Heim, M. A. , Jakoby, M. , Werber, M. , Martin, C. , Weisshaar, B. , & Bailey, P. C. (2003). The basic Helix‐Loop‐Helix transcription factor family in plants: A genome‐wide study of protein structure and functional diversity. Molecular Biology and Evolution, 20(5), 735–747. 10.1093/molbev/msg088 12679534

[ppp310132-bib-0024] James, A. M. , Ma, D. , Mellway, R. D. , Gesell, A. , Yoshida, K. , Walker, V. , … Peter Constabel, C. (2017). MYB115 and MYB134 transcription factors regulate proanthocyanidin synthesis and structure. Plant Physiology, 174(1). 10.1104/pp.16.01962 PMC5411147 28348066

[ppp310132-bib-0025] Junk‐Knievel, D. C. , Vandenberg, A. , & Bett, K. E. (2007). An accelerated postharvest seed‐coat darkening protocol for pinto beans grown across different environments. Crop Science, 47, 694–702. 10.2135/cropsci2006.05.0325

[ppp310132-bib-0026] Junk‐Knievel, D. C. , Vandenberg, A. , & Bett, K. E. (2008). Slow darkening in pinto bean (*Phaseolus vulgaris* L.) *s*eed coats is controlled by a single major gene. Crop Science, 48(1), 189–193.

[ppp310132-bib-0027] Kong, Q. , Pattanaik, S. , Feller, A. , Werkman, J. R. , Chai, C. , Wang, Y. , … Yuan, L. (2012). Regulatory switch enforced by basic Helix‐Loop‐Helix and ACT‐domain mediated dimerizations of the maize transcription factor R. Proceedings of the National Academy of Sciences of the United States of America, 109(30), 2091–2097. 10.1073/pnas.1205513109 PMC3409794 22778424

[ppp310132-bib-0028] Kumar, S. , Stecher, G. , & Tamura, K. (2016). MEGA7: Molecular evolutionary genetics analysis version 7.0 for bigger datasets. Molecular Biology and Evolution, 33(7), 1870–1874. 10.1093/molbev/msw054 27004904 PMC8210823

[ppp310132-bib-0029] Li, P. , Chen, B. , Zhang, G. , Chen, L. , Dong, Q. , Wen, J. , … Zhao, J. (2016). Regulation of anthocyanin and proanthocyanidin biosynthesis by *Medicago truncatula* bHLH transcription factor MtTT8. New Phytologist, 210(3), 905–921. 10.1111/nph.13816 26725247

[ppp310132-bib-0030] Li, X. , Chen, L. I. , Hong, M. , Zhang, Y. , Zu, F. , Wen, J. , … Fu, T. (2012). A large insertion in bHLH transcription factor *BrTT8* resulting in yellow seed coat in *Brassica rapa* . PLoS One, 7(9), e44145. 10.1371/journal.pone.0044145 22984469 PMC3439492

[ppp310132-bib-0031] Li, Y. G. , Tanner, G. , & Larkin, P. (1996). The DMACA–HCl protocol and the threshold proanthocyanidin content for bloat safety in forage legumes. Journal of the Science of Food and Agriculture, 70(1), 89–101. 10.1002/(SICI)1097-0010(199601)70:1<89:AID-JSFA470>3.0.CO;2-N

[ppp310132-bib-0032] Liu, C. , Jun, J. H. , & Dixon, R. A. (2014). MYB5 and MYB14 play pivotal roles in seed coat polymer biosynthesis in *Medicago truncatula* . Plant Physiology, 165(4), 1424–1439. 24948832 10.1104/pp.114.241877 PMC4119029

[ppp310132-bib-0033] Liu, C. , Wang, X. , Shulaev, V. , & Dixon, R. A. (2016). A role for leucoanthocyanidin reductase in the extension of proanthocyanidins. Nature Plants, 2(12), 16182. 10.1038/nplants.2016.182 27869786

[ppp310132-bib-0034] Lu, N. , Roldan, M. , & Dixon, R. A. (2017). Characterization of two TT2‐type MYB transcription factors regulating proanthocyanidin biosynthesis in tetraploid cotton, *Gossypium hirsutum* . Planta, 246(2), 323–335. 10.1007/s00425-017-2682-z 28421329

[ppp310132-bib-0035] McClean, P. E. , Bett, K. E. , Stonehouse, R. , Lee, R. , Pflieger, S. , Moghaddam, S. M. , … Mamidi, S. (2018). White seed color in common bean (*Phaseolus vulgaris*) results from convergent evolution in the *P* (pigment) gene. New Phytologist, 219(2), 1–12. 10.1111/nph.15259 29897103

[ppp310132-bib-0036] McClean, P. E. , Lee, R. K. , Otto, C. , Gepts, P. , & Bassett, M. J. (2002). Molecular and phenotypic mapping of genes controlling seed coat pattern and color in common bean (*Phaseolus vulgaris* L.). Journal of Heredity, 93(2), 148–152. 10.1093/jhered/93.2.148 12140276

[ppp310132-bib-0037] Murray, M. G. , & Thompson, W. F. (1980). Rapid isolation of high molecular weight plant DNA. Nucleic Acids Research, 8(19), 4321–4325. 10.1093/nar/8.19.4321 7433111 PMC324241

[ppp310132-bib-0038] Nesi, N. , Debeaujon, I. , Jond, C. , Pelletier, G. , Caboche, M. , & Lepiniec, L. (2000). The *TT8* gene encodes a basic Helix‐Loop‐Helix domain protein required for expression of *DFR* and *BAN* genes in Arabidopsis siliques. The Plant Cell, 12, 1863–1878. 11041882 10.1105/tpc.12.10.1863 PMC149125

[ppp310132-bib-0039] Nesi, N. , Jond, C. , Debeaujon, I. , Caboche, M. , & Lepiniec, L. (2001). The *Arabidopsis TT2* gene encodes an R2R3 MYB domain protein that acts as a key determinant for proanthocyanidin accumulation in developing seed. The Plant Cell, 13, 2099–2114. 11549766 10.1105/TPC.010098 PMC139454

[ppp310132-bib-0040] Onufriev, A. V. , & Alexov, E. (2013). Protonation and pK changes in protein‐ligand binding. Quarterly Reviews of Biophysics, 46(2), 181–209. 10.1017/S0033583513000024 23889892 PMC4437766

[ppp310132-bib-0041] Osorno, J. M. , Grafton, K. F. , Rojas‐Cifuentes, G. A. , Gelin, R. , & Vander Wal, A. J. (2010). Registration of ‘Lariat’ and ‘Stampede’ pinto beans. Journal of Plant Registrations, 4(1), 5–11. 10.3198/jpr2009.03.0143crc

[ppp310132-bib-0042] Osorno, J. M. , Vander Wal, A. J. , Kloberdanz, M. , Pasche, J. S. , Schroder, S. , & Miklas, P. N. (2018). A new Slow‐darkening pinto bean with improved agronomic performance: Registration of ‘ND‐Palomino’. Journal of Plant Registrations, 12, 25–30. 10.3198/jpr2017.05.0026crc

[ppp310132-bib-0043] Pang, Y. , Peel, G. J. , Sharma, S. B. , Tang, Y. , & Dixon, R. A. (2008). A transcript profiling approach reveals an epicatechin‐specific glucosyltransferase expressed in the seed coat of *Medicago truncatula* . Proceedings of the National Academy of Sciences of the United States of America, 105(37), 14210–14215. 10.1073/pnas.0805954105 18772380 PMC2544603

[ppp310132-bib-0044] Park, D. , & Maga, J. A. (1999). Dry bean (*Phaseolus vulgaris*) color stability as influenced by time and moisture content. Journal of Food Processing Preservation, 23, 515–522.

[ppp310132-bib-0045] Pourcel, L. , Routaboul, J. M. , Cheynier, V. , Lepiniec, L. , & Debeaujon, I. (2007). Flavonoid oxidation in plants: From biochemical properties to physiological functions. Trends in Plant Science, 12, 29–36. 10.1016/j.tplants.2006.11.006 17161643

[ppp310132-bib-0046] Ramsay, N. A. , & Glover, B. J. (2005). MYB‐bHLH‐WD40 protein complex and the evolution of cellular diversity. Trends in Plant Science, 10(2), 63–70. 10.1016/j.tplants.2004.12.011 15708343

[ppp310132-bib-0047] Roth, B. A. , Goff, S. A. , Klein, T. M. , & Fromm, M. E. (1991). C1‐ and R‐dependent expression of the maize *Bz1* gene requires sequences with homology to mammalian MYB and MYC binding sites. The Plant Cell, 3(3), 317–325. 1840914 10.1105/tpc.3.3.317 PMC160002

[ppp310132-bib-0048] Sanchez‐Valdez, I. , Acosta‐Gallegos, J. , Ibarra‐Pérez, F. , Rosales‐Serna, R. , & Singh, S. (2004). Registration of ‘Pinto Saltillo’ common bean. Crop Science, 44. 10.2135/cropsci2004.1865a

[ppp310132-bib-0049] Schmutz, J. , McClean, P. E. , Mamidi, S. , Wu, G. A. , Cannon, S. B. , Grimwood, J. , … Jackson, S. A. (2014). A reference genome for common bean and genome‐wide analysis of dual domestications. Nature Genetics, 46, 707–713. 10.1038/ng.3008 24908249 PMC7048698

[ppp310132-bib-0050] Shirley, B. W. , Kubasek, W. L. , Storz, G. , Bruggemann, E. , Koornneef, M. , Ausubel, F. M. , & Goodman, H. M. (1995). Analysis of Arabidopsis mutants deficient in flavonoid biosynthesis. The Plant Journal, 8, 659–671. 10.1046/j.1365-313X.1995.08050659.x 8528278

[ppp310132-bib-0051] Sievers, F. , Wilm, A. , Dineen, D. , Gibson, T. J. , Karplus, K. , Li, W. , … Söding, J. et al (2011). Fast, scalable generation of high‐quality protein multiple sequence alignments using Clustal Omega. Molecular Systems Biology, 7(1), 539–545. 21988835 10.1038/msb.2011.75 PMC3261699

[ppp310132-bib-0052] Sparkes, I. A. , Runions, J. , Kearns, A. , & Hawes, C. (2006). Rapid, transient expression of fluorescent fusion proteins in tobacco plants and generation of stably transformed plants. Nature Protocols, 1(4), 2019–2025. 10.1038/nprot.2006.286 17487191

[ppp310132-bib-0053] Taylor, L. P. , & Briggs, W. R. (1990). Genetic regulation and photocontrol of anthocyanin accumulation in maize seedlings. The Plant Cell, 2(2), 115–127. 2136630 10.1105/tpc.2.2.115 PMC159869

[ppp310132-bib-0054] Vernoud, V. , Horton, A. C. , Yang, Z. , & Nielsen, E. (2003). Analysis of the small GTPase gene superfamily of *Arabidopsis* . Plant Physiology, 131(3), 1191–1208. 10.1104/pp.013052 12644670 PMC166880

[ppp310132-bib-0055] Wahl, V. , Brand, L. H. , Guo, Y. L. , & Schmid, M. (2010). The FANTASTIC FOUR proteins influence shoot meristem size in *Arabidopsis thaliana* . BMC Plant Biology, 10, 285. 10.1186/1471-2229-10-285 21176196 PMC3023791

[ppp310132-bib-0056] Walker, A. R. , Davison, P. A. , Bolognesi‐Winfield, A. C. , James, C. M. , Srinivasan, N. , Blundell, T. L. , … Gray, J. C. (1999). The *TRANSPARENT TESTA GLABRA1* locus, which regulates trichome differentiation and anthocyanin biosynthesis in *Arabidopsis*, encodes a WD40 repeat protein. The Plant Cell, 11(7), 1337–1349. 10402433 10.1105/tpc.11.7.1337 PMC144274

[ppp310132-bib-0057] Xu, W. , Dubos, C. , & Lepiniec, L. (2015). Transcriptional control of flavonoid biosynthesis by MYB–bHLH–WDR complexes. Trends in Plant Science, 20(3), 176–185. 10.1016/j.tplants.2014.12.001 25577424

[ppp310132-bib-0058] Zhang, B. , & Schrader, A. (2017). TRANSPARENT TESTA GLABRA 1‐dependent regulation of flavonoid biosynthesis. Plants, 6(4), 65–95. 10.3390/plants6040065 PMC5750641 29261137

[ppp310132-bib-0059] Zhao, J. , & Dixon, R. A. (2009). MATE transporters facilitate vacuolar uptake of epicatechin 3'‐O‐glucoside for proanthocyanidin biosynthesis in *Medicago truncatula* and *Arabidopsis* . The Plant Cell, 21, 2323–2340. 19684242 10.1105/tpc.109.067819 PMC2751950

[ppp310132-bib-0060] Zhao, J. , Pang, Y. , & Dixon, R. A. (2010). The mysteries of proanthocyanidin transport and polymerization. Plant Physiology, 153, 437–443. 10.1104/pp.110.155432 20388668 PMC2879784

