# Supplementary material for: Postharvest seed coat darkening in pinto bean (Phaseolus vulgaris) is regulated by Psd, an allele of the basic helix‐loop‐helix transcription factor P
Source: Plants People Planet. 2020 Aug 19;2(6):663–77. doi: 10.1002/ppp3.10132 (PMC8262261; doi:10.1002/ppp3.10132)
Supplement: Supplementary file 1 — Supplementary Material [file PPP3-2-663-s001.pdf]

***Plants, People, Planet* Supporting Information**

Article title: Postharvest seed coat darkening in pinto bean (*Phaseolus vulgaris*) is regulated by P^{sd}, an allele of the basic helix-loop-helix transcription factor *P*.

Authors: Nishat S. Islam, Kirstin E. Bett, K. Peter Pauls, Frédéric Marsolais and Sangeeta Dhaubhadel

The following Supporting Information is available for this article:

Table S1 List of pinto bean genotypes and sources

Table S2 List of primers used for cloning, gene expression and gene identification

Fig. S1 Protein sequence alignment of P^{sd}-1 with other characterized TT8s.

Fig. S2 Gene structure and phylogenetic analysis of P.

Fig. S3 Protein sequence comparisons of P^{sd} isoforms.

Fig. S4 Presence of transcript variants *P-1, P^{sd}-1, P-2* and *P^{sd}-2* in pinto bean.

Fig. S5 Seed coat phenotype of Arabidopsis seeds in multiple complementation lines.

Fig. S6 Transcript and metabolite accumulation in Arabidopsis T2 complementation lines.

Fig. S7 Genetics of *J* and *Sd* alleles.

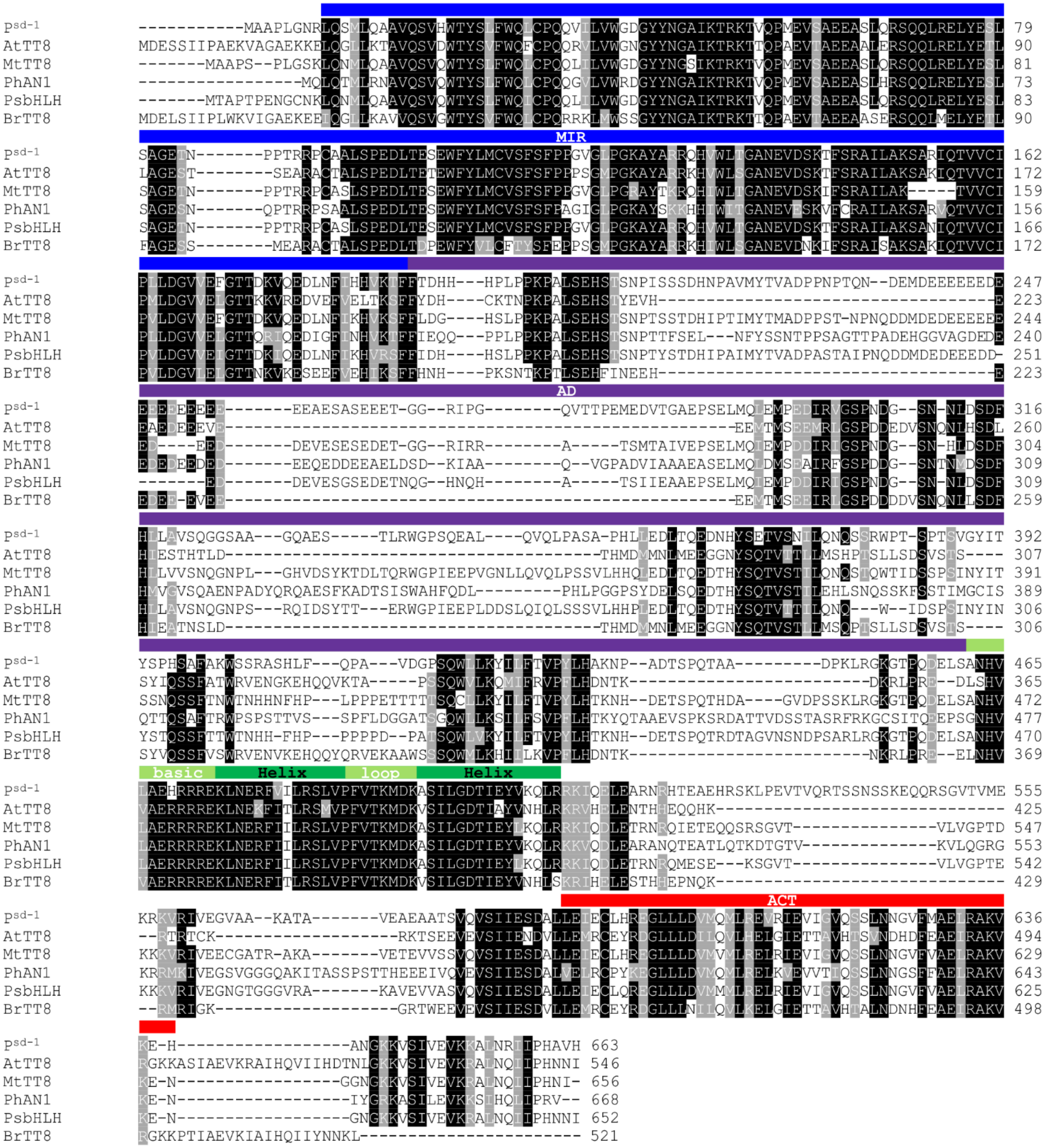

Fig. S1 Protein sequence alignment of P^{sd}-1 with other characterized TT8s. Coloured bars above the sequences indicate protein motifs as in Fig. 4b.

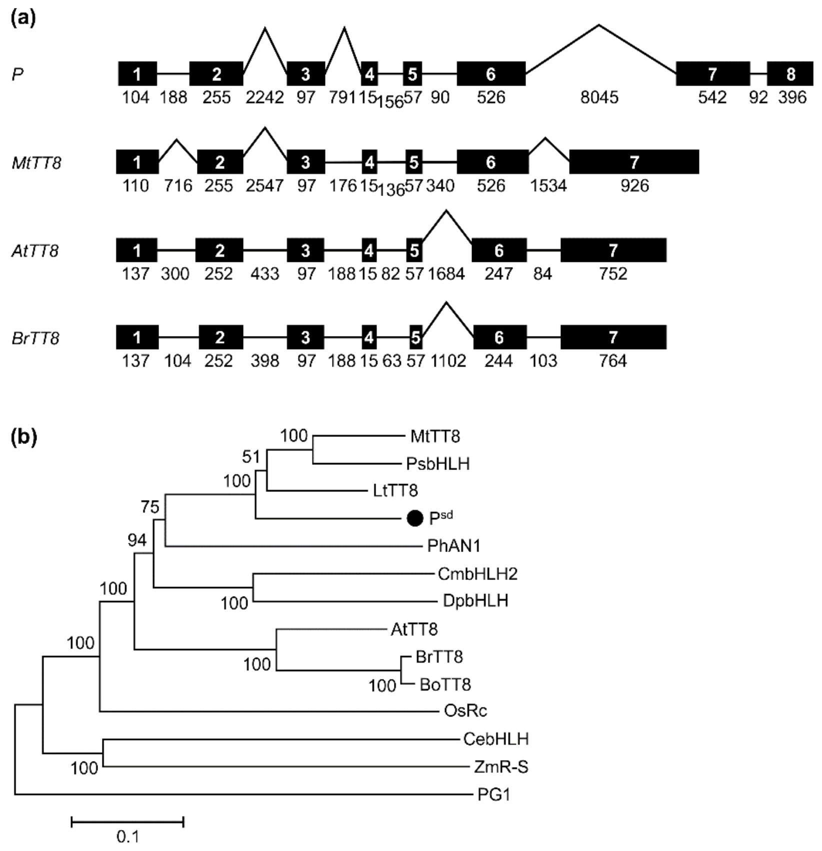

Fig. S2 Gene structure and phylogenetic analysis of P. (a) Gene structure of *P* and characterized *TT8* genes from other plant species. Black rectangular boxes with numbers represent exons and lines represent introns (not drawn to scale). Numbers indicate the sizes of exons and introns in nucleotides (bp). Accession numbers: Arabidopsis *AtTT8* (NM_117050), *M. truncatula MtTT8* (KM892777), *B. rapa BrTT8* (ABY59772.1). (b) Protein sequence of P^{sd} and TT8 from other plant species were aligned using ClustalO and phylogenetic tree was constructed by neighbour-joining method using MEGA7. Bootstrap values (1000 replicates) are shown as percentages next to branch points. Accession numbers: Arabidopsis AtTT8 (NP_192720.2), *Brassica oleracea* BoTT8 (ADP76654.1), *B. rapa* BrTT8 (AEA03281), *Chrysanthemum* × *morifolium* CmbHLH2 (ARK19321.1), *Cornus eydeana* CebHLH (AAR21675.1), *Dahlia pinnata* DpbHLH (BAM84241.1), *Lotus tenuis* LtTT8 (ALR72603.1), *M. truncatula* MtTT8 (AKN79606), *Oryza sativa* OsRc (AB247503), *P. vulgaris* P^{sd} (Phvul.007G171333), *Petunia* × *hybrida* PhAN1 (AAG25927.1), *Pisum sativum* PsbHLH (ADO13282.1), *Zea mays* ZmR-S (NP_001105339.2). PG1 (*P. vulgaris*, Phaseolin G-box 1) is included as outgroup.

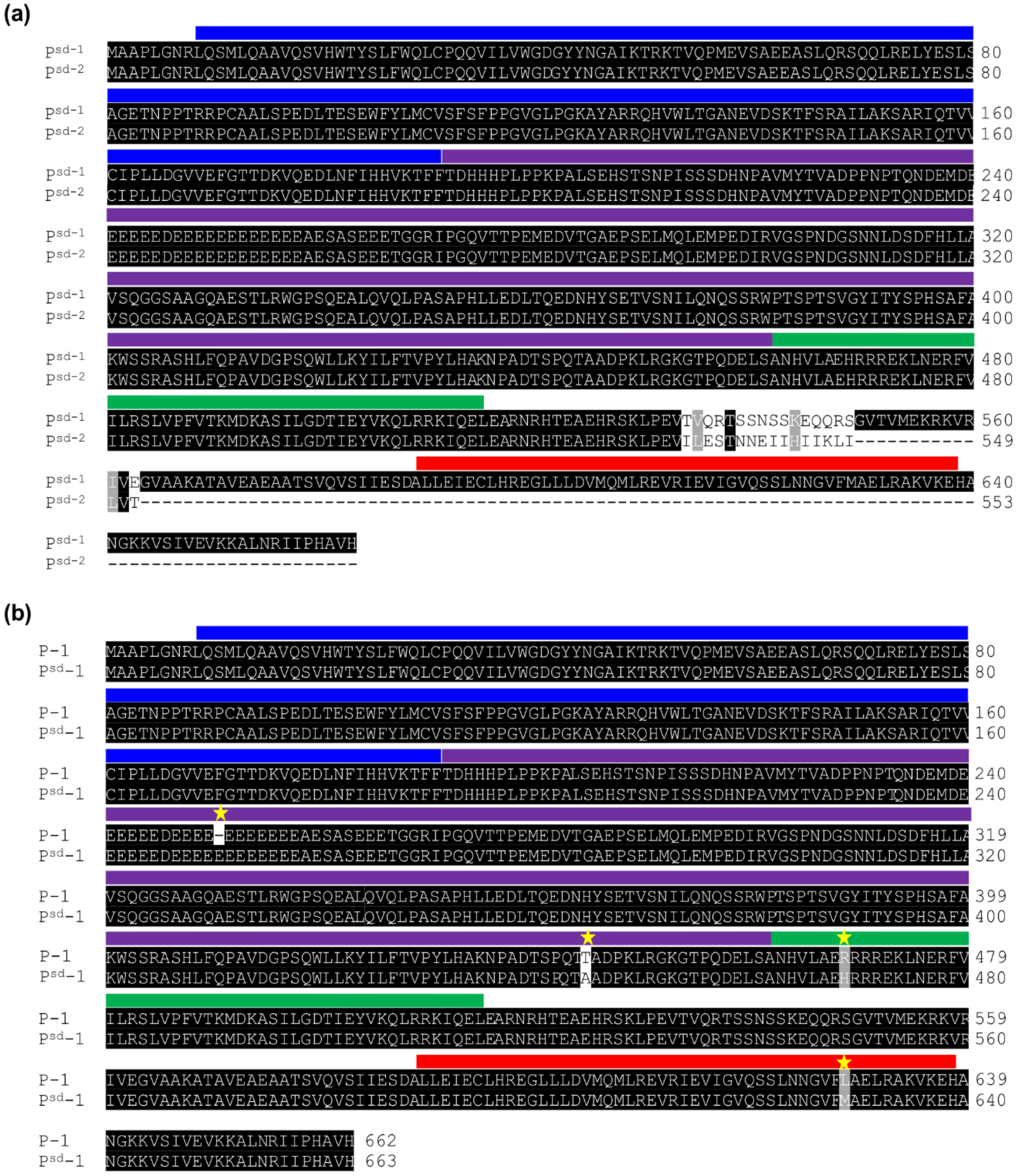

Fig. S3 Protein sequence comparisons of P^{sd} isoforms. (a) Alignment of predicted protein sequences encoded by two transcript variants *P^{sd}-1* and *P^{sd}-2* in pinto bean. Colours indicative of protein motifs as in Fig 4b. (b) Alignment of predicted protein sequences encoded by *P-1* and *P^{sd}-1* from CDC Pintium and 1533-15, respectively.

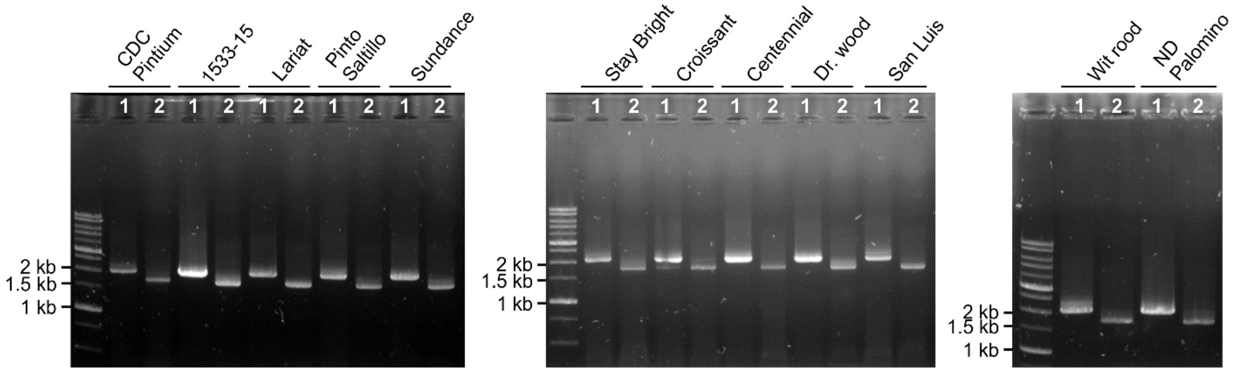

Fig. S4 Presence of transcript variants *P-1*, P^{sd}*-1*, *P-2* and P^{sd}*-2* in pinto bean. Total RNA isolated from seed tissue collected from 21 different pinto bean lines were used for cDNA synthesis and RT-PCR using the transcript-specific primers as mentioned in Fig 4a. 1, *P-1* or P^{sd}*-1*; 2, *P-2* or P^{sd}*-2*.

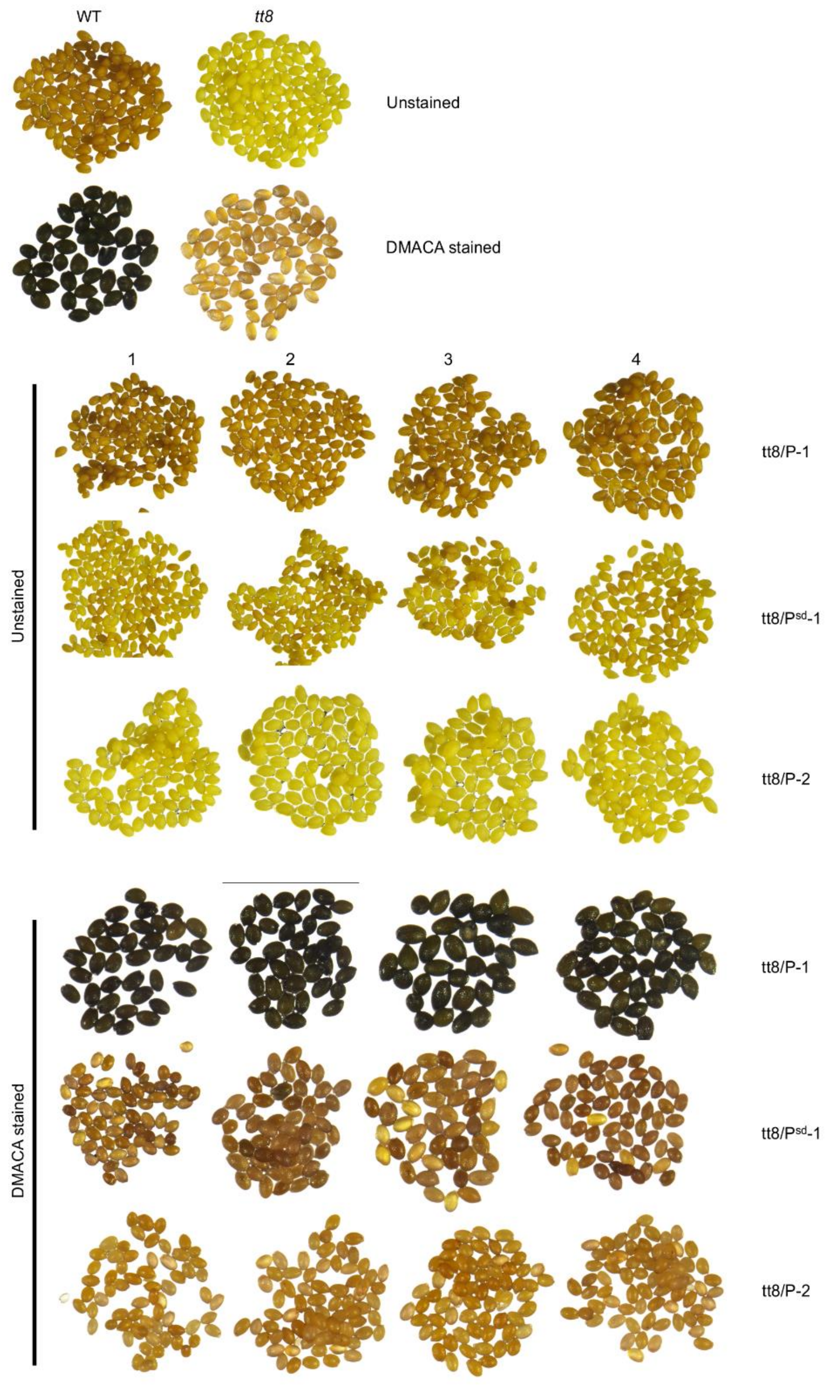

Fig. S5 Seed coat phenotype of Arabidopsis seeds in multiple complementation lines. Arabidopsis *tt8* mutant line was transformed with *P* and P^{sd} transcript variants from CDC Pintium and 1533-15, respectively. Photographs of unstained and DMACA stained seeds of wild-type (WT), *tt8* and four independent T2 transgenic lines for tt8/P-1-CDC Pintium, tt8/P^{sd}-1-1533-15 and tt8/P-2-CDC Pintium are shown. Numbers indicate independent lines.

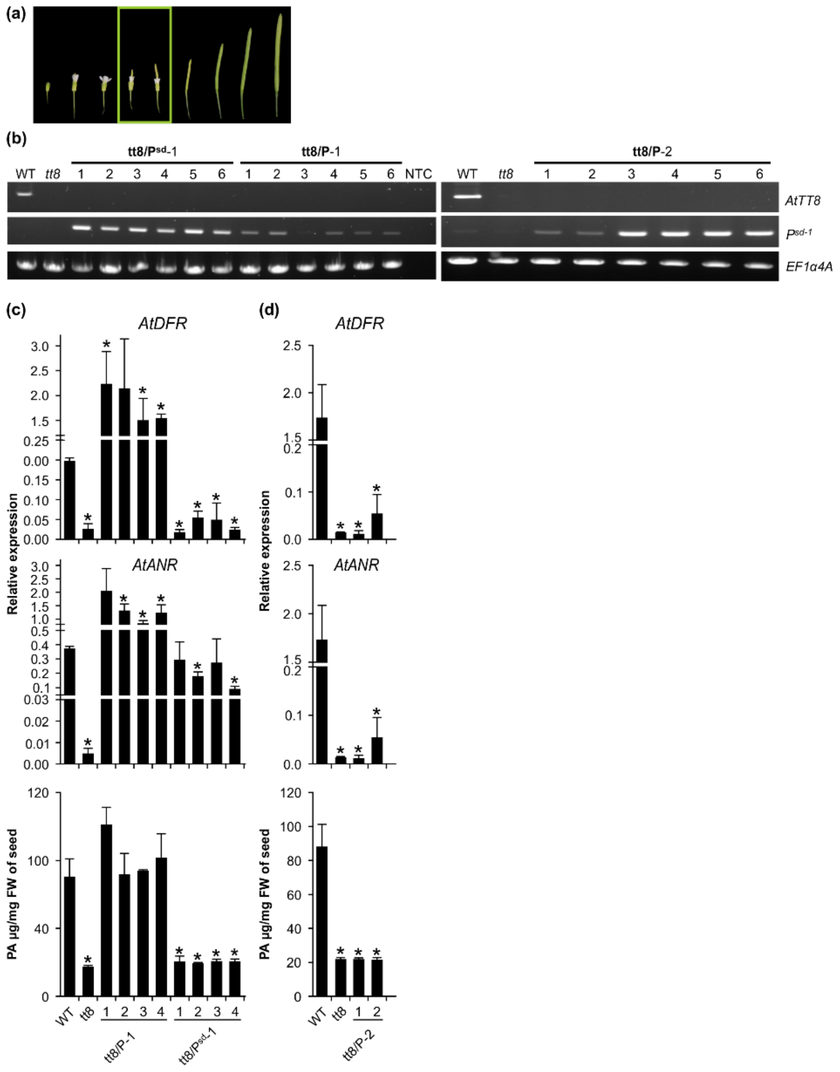

Fig. S6 Transcript and metabolite accumulation in Arabidopsis T2 complementation lines. (a) A photograph showing developmental stages of siliques in Arabidopsis. Green box indicates the stage of siliques collected from Arabidopsis T1 complementation lines for RNA extraction and gene expression study. (b) RT-PCR to confirm mutant background and expression of *P* or P^{sd} in seeds from multiple T2 complementation lines. (c) Accumulation of *AtDFR* and *AtANR* transcripts and PA in wild-type (WT), *tt8*, and in 4 individual lines of tt8/P-1-CDC Pintium and tt8/P^{sd}-1-1533-15. (d) Accumulation of *AtDFR* and *AtANR* transcripts and PA in WT, *tt8*, and in 2 individual lines of tt8/P-2-CDC Pintium. All gene expression analysis was normalized with ubiquitin and PA accumulation was compared with proanthocyanidin A2 standard. Asterisks (*) indicate significant difference with respect to WT at $P < 0.05$ by Student's *t*-test.

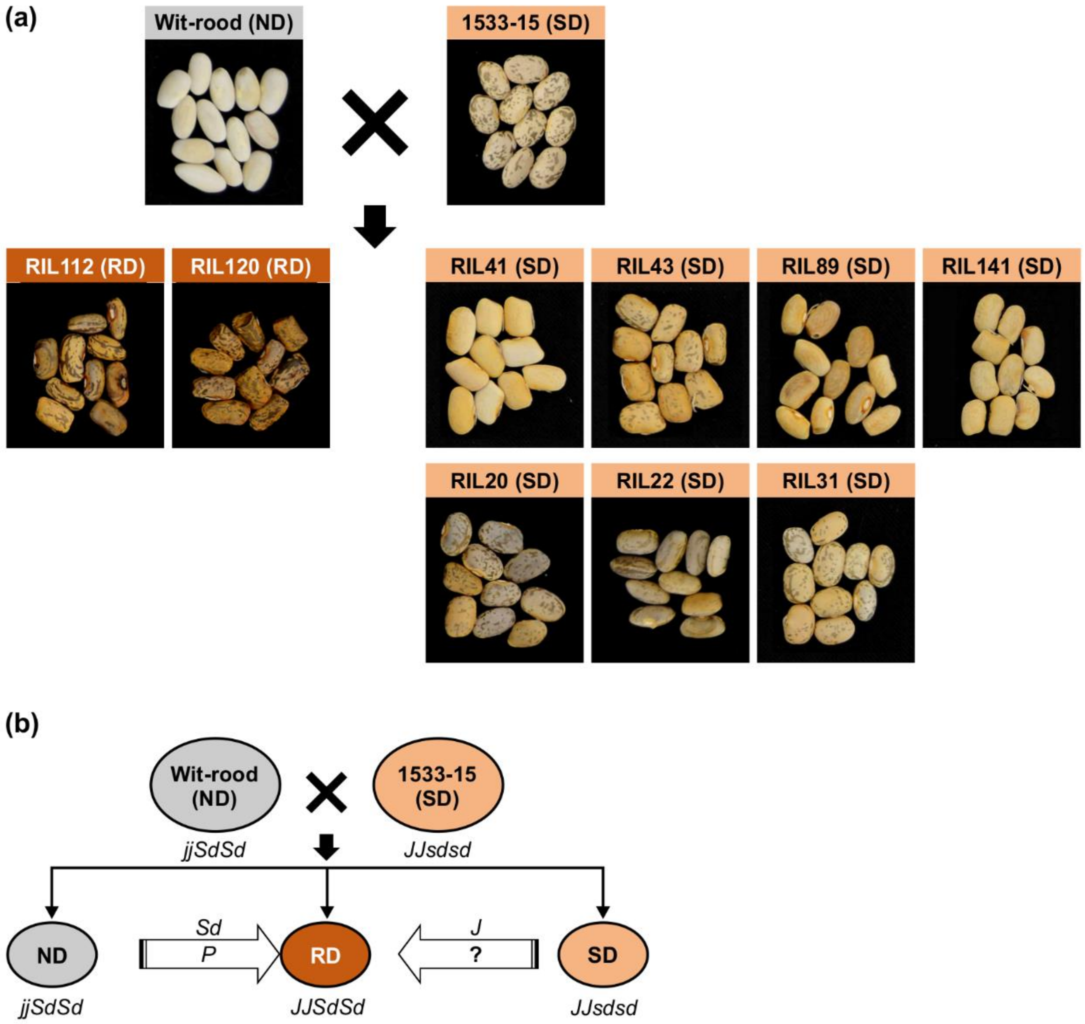

Fig. S7 Genetics of *J* and *Sd* alleles. (a) Postharvest seed phenotype showing SD and RD recombinant inbred lines (RILs) derived from cross between 1533-15 (SD) and Wit-rood boontje (ND). ND is not shown. (b) A prospective pattern of inheritance of *J* and *Sd* in the RILs and the epistatic nature of *J* on *Sd* in seed coat postharvest darkening.

Table S1 List of pinto bean genotypes and sources

#	Cultivars/RILs	Postharvest seed coat color trait	Source
1	CDC Pintium	RD	Dr. Kirstin Bett, University of Saskatchewan, Canada
2	1533-15	SD	
3	Sundance	SD	Dr. Mark A. Brick, Colorado State University, USA
4	StayBright	SD	
5	Croissant	RD	
6	Centennial	RD	
7	DR Wood	RD	
8	Lariat	RD	Dr. Juan M. Osorno, North Dakota State University, USA
9	ND Palomino	SD	
10	Pinto Saltillo	SD	Dr. Jorge Alberto Acosta-Gallegos, Instituto Nacional de Investigaciones Forestales, Agrícolas y Pecuarias (INIFAP) Celaya, Mexico
11	San Luis	RD	
12	Wit-rood boontje	ND	Dr. Peter Pauls, University of Guelph, Canada
13	RIL20	SD	
14	RIL22	SD	
15	RIL31	SD	
16	RIL41	SD	
17	RIL43	SD	
18	RIL89	SD	
19	RIL141	SD	
20	RIL112	RD	
21	RIL120	RD	

Table S2 List of primers used for cloning, gene expression and gene identification

Primer names	Sequences (5' to 3')	Purpose	
**bHLH333L-GWF**	GGGGACAAGTTTGTACAAAAAAGCAGGCTTCATGGCTGCACCACTAGGCAATA	*P-1*, P^{sd}*-1*	ORF Cloning
**bHLH333L-GWR**	GGGGACCACTTTGTACAAGAAAGCTGGGTCGTGGACAGCGTGGGGAATGATTCG		
**bHLH333S-GWF**	GGGGACAAGTTTGTACAAAAAAGCAGGCTTCATGGCTGCACCACTAGGCAATA	*P-2*, P^{sd}*-2*	
**bHLH333S-GWR**	GGGGACCACTTTGTACAAGAAAGCTGGGTCTGTCACCAAAATCAACTTTATA		
**deb122RB1**	AGGAAGACAACTCAACCAGC	*tt8* genotyping (Nesi *et al.*, 2000)	
**deb122LB2**	TCATCAGAATACAATTCTCAAATCT		
**deb122LB3**	CTCCACGTGGCAAACGATGATTGG		
**qbHLHF1**	AAGATCCAGGAGCTGGAGGC	*P*	Gene expression
**qbHLHR1**	CCTTGCTCGAATTGCTACTCGTCC		
**AtDFRF**	ATGGTTAGTCAGAAAGAGACCG	*AT5G42800.1*	
**AtDFRR**	GTCTTATGATCGAGTAATGCGC		
**qAtDFRR**	TAGCCATGAACCGATGAAACC		
**AtANRF**	AACAACTAAATCTCTATCTCTGTA	*AF092912.1*	
**AtANRR**	GAATGAGACCAAAGACTCATATAC		
**qAtANRF**	GGACTGACATTGATTTTCTCACAG		
**qAtANRR**	TGCTAGCACCTTCGAGATTG		
**AtEF1αA4F**	ATGCCCCAGGACATCGTGATTTCAT	*X16432*	
**AtEF1αA4R**	TTGGCGGCACCCTTAGCTGGATCA		
**qAtUBQF**	GTGGTGCTAAGAAGAGGAAGA	*AT3G62250.1*	
**qAtUBQR**	TCAAGCTTCAACTCCTTCTTT		
**PvbHLHp12804F**	AGCTGAGCGCCAACCA	SNP marker PvsdSNP	
**PvbHLHp12804R**	ACCGCTCGTTCAGCTTCTC		
**PvindelF**	GAC CCA CCC AAT CCT ACC C	InDel marker PvsdInDel	
**PvindelR**	CCTCCGGTTTCTTCCTCAGA		
